# Supplementary material for: Real-Time Ligand Binding of Fluorescent VEGF-A Isoforms that Discriminate between VEGFR2 and NRP1 in Living Cells
Source: Cell Chem Biol. 2018 Oct 18;25(10):1208–1218.e5. doi: 10.1016/j.chembiol.2018.06.012 (PMC6200776; doi:10.1016/j.chembiol.2018.06.012)
Supplement: Document S1. Figures S1–S4 and Tables S1–S3 [file mmc1.pdf]

Cell Chemical Biology, Volume *25*

Supplemental Information

Real-Time Ligand Binding of Fluorescent VEGF-A Isoforms that Discriminate between VEGFR2 and NRP1 in Living Cells

Chloe J. Peach, Laura E. Kilpatrick, Rachel Friedman-Ohana, Kris Zimmerman, Matthew B. Robers, Keith V. Wood, Jeanette Woolard, and Stephen J. Hill

Supplementary Information.

Real-time ligand binding to VEGFR2 and Neuropilin-1 with fluorescent VEGF-A isoforms that discriminate between the two cell surface proteins in living cells

Chloe J. Peach[1,2,♯], Laura E. Kilpatrick[1,2,♯], Rachel Friedman-Ohana[3], Kris Zimmerman[3], Matthew B. Robers[3], Keith V. Wood[3], Jeanette Woolard[1,2,*], Stephen J. Hill[1,2,*]

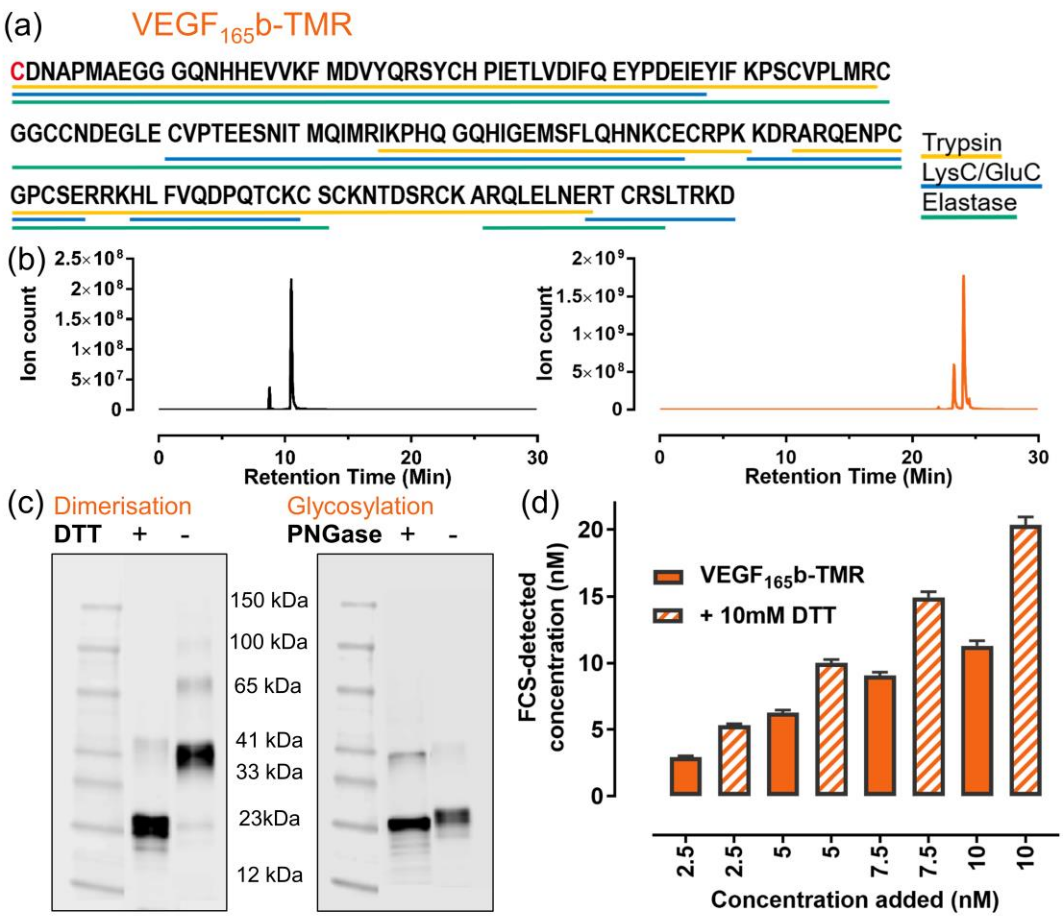

Figure S1. LC-MS/MS analysis of VEGF$_{165}$b-TMR digested with multiple proteases. (Relates to Figure 1). (a) Peptide coverage achieved by digestion with trypsin, LysC/GluC and elastase proteases. The N-terminal cysteine is marked in red. None of the other 14 residues presented in the VEGF$_{165}$b protomer were labelled. Protein identity was confirmed by searching the MS/MS spectra using the Mascot search engine (Matrix Science Inc, Boston, USA) against a human database (SwissProt). The highest scoring hit was the VEGF sequence. (b) LC-MS/MS analysis of the peptide containing the N terminal cysteine (CDNAPMAEGGGQNHHEVVK) derived from VEGF$_{165}$b-TMR (right panel) and VEGF$_{165}$b (left panel) that were purified in the same manner and digested with trypsin protease. Retention times and mono-isotopic masses are given in Supplementary Table 1. (c) Fluorescence SDS-PAGE analysis of VEGF$_{165}$b-TMR (E_{ex}=532 nm; E_{em}=580 nm) in the presence or absence of 100mM DTT and with or without deglycosylation by PNGase. (d) VEGF$_{165}$b-TMR concentration determined using fluorescence correlation spectroscopy (FCS) in the presence or absence of 10mM DTT. N=4-6 separate experiments.

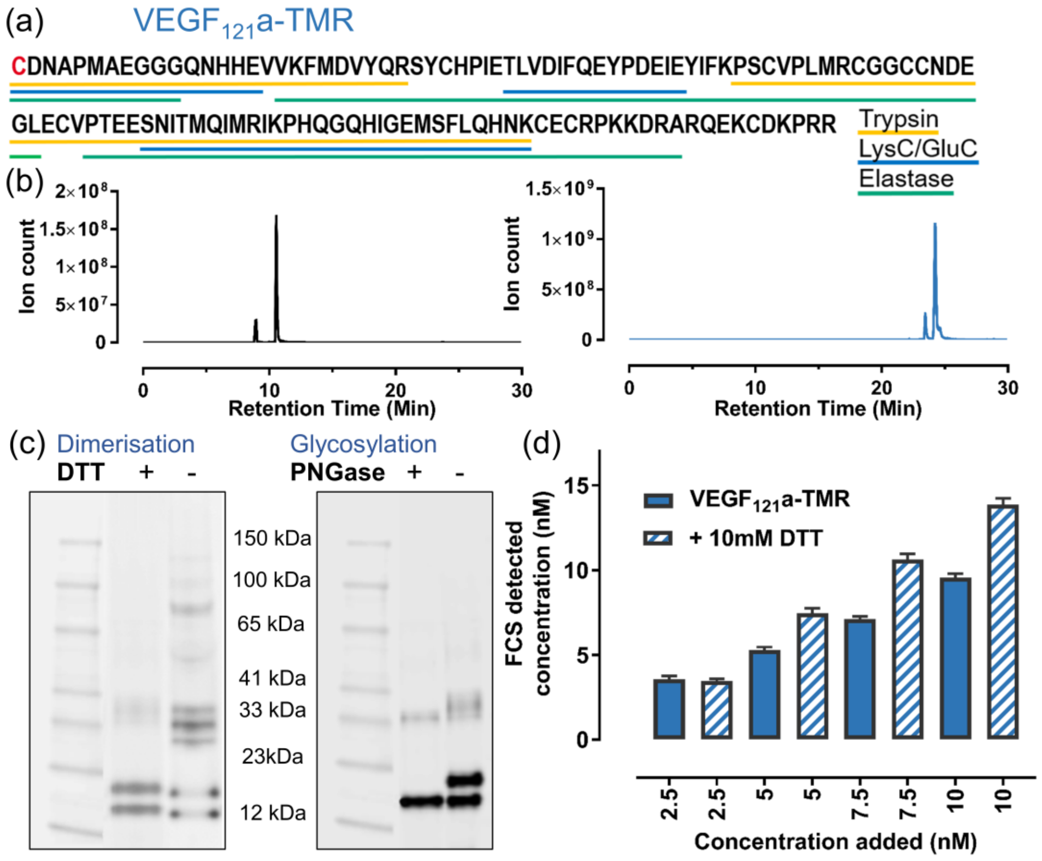

Figure S2. LC-MS/MS analysis of VEGF$_{121}$a-TMR digested with multiple proteases. (Relates to Figure 1). (a) Peptide coverage achieved by digestion with trypsin, LysC/GluC and elastase proteases. The N-terminal cysteine is marked in red. None of the other 8 residues presented in the VEGF$_{121}$a protomer were labelled. Protein identity was confirmed by searching the MS/MS spectra using the Mascot search engine (Matrix Science Inc, Boston, USA) against a human database (SwissProt). The highest scoring hit was the VEGF sequence. (b) LC-MS/MS analysis of the peptide containing the N terminal cysteine (CDNAPMAEGGGQNHHEVVK) derived from VEGF$_{121}$a-TMR (right panel) and VEGF$_{121}$a (left panel) that were purified in the same manner and digested with trypsin protease. Retention times and mono-isotopic masses are given in Supplementary Table 1. (c) Fluorescence SDS-PAGE analysis of VEGF$_{121}$a-TMR (E_{ex}=532 nm; E_{em}=580 nm) in the presence or absence of 100mM DTT and with or without deglycosylation by PNGase. (d) VEGF$_{121}$a-TMR concentration in the presence or absence of 10mM DTT quantified using fluorescence correlation spectroscopy (FCS). N=3-4 separate experiments.

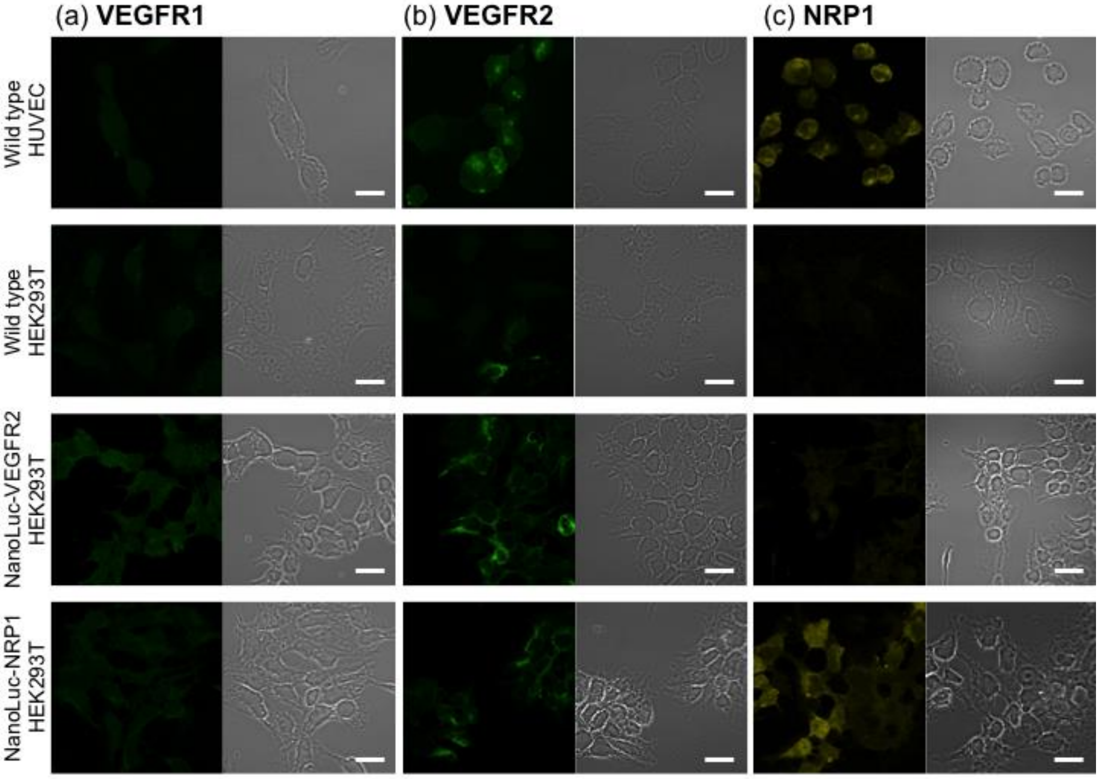

Figure S3. Immunofluorescence labelling of VEGFR1, VEGFR2 and Neuropilin-1 (NRP1) expression in HEK293 cells and HUVECs. (Relates to Figure 2). Immunofluorescence staining for either (A) VEGFR1 (green), (B) VEGFR2 (green) or (B) Neuropilin-1 (yellow), alongside phase contrast images, in fixed cells on coverslips imaged using the Zeiss Confocal LSM880 (63X magnification). Scale bars shown as 20μm and images are representative of n=3.

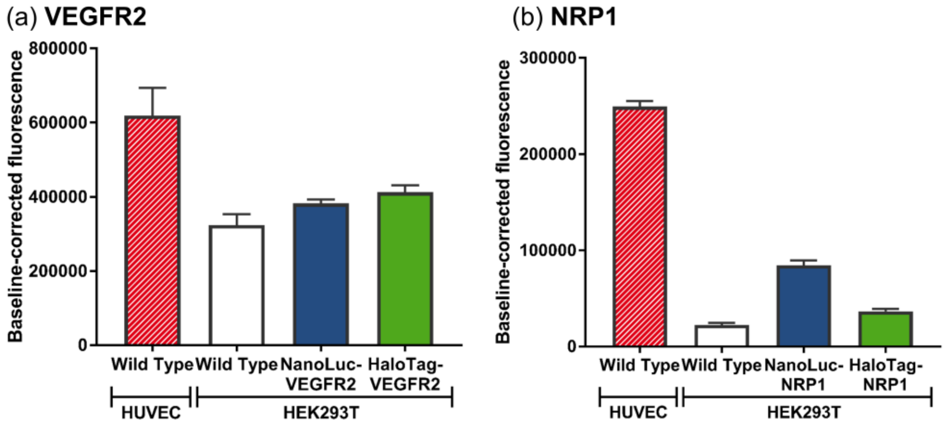

Figure S4. Relative expression of VEGFR2 and NRP1 in endothelial and transfected cell lines. (Relates to Figure 2). Quantified immunofluorescence staining for (A) VEGFR2 or (B) Neuropilin-1, in fixed HUVEC or HEK293T cells imaged in 96-well plates using ImageXpress Micro (20x magnification). Fluorescence was quantified on a per cell basis and baseline-corrected for non-specific fluorescence per experiment (secondary antibody only). Data shown as mean±S.E.M. from 5 independent experiments.

Table S1. (Relates to Figure 1).

LS-MS/MS profiles for unlabelled peptide fragments obtained following protease digestion of VEGF or VEGF-TMR.

**Peptide sequence**	**Digest**	**VEGF isoform**	**Mass/charge (m/z)**	**Charge (z)**	**Mass [M+H]***	**Retention Time (Min)**	**Peak Area**	**Sample**	**%**	**% Labelled**
CDNAPMAEGGGQNHHEVVK	Trypsin	$VEGF_{165}b$	498.97	4	1992.89	10.5	1.36E+08	Labelled	2.9	97.1
CDNAPMAEGGGQNHHEVVK	Trypsin	$VEGF_{165}b$	498.97	4	1992.89	10.5	4.66E+09	Unlabelled	100	n/a
CDNAPMAEGGGQNHHEVVK	Trypsin	$VEGF_{121}a$	498.97	4	1992.89	10.5	6.20E+08	Labelled	6.1	93.9
CDNAPMAEGGGQNHHEVVK	Trypsin	$VEGF_{121}a$	498.97	4	1992.89	10.3	1.01E+10	Unlabelled	100	n/a
CDNAPMAE	LysC/GluC	$VEGF_{165}b$	425.66	2	850.31	13.7	5.26E+06	Labelled	3.5	96.5
CDNAPMAE	LysC/GluC	$VEGF_{165}b$	425.66	2	850.31	13.6	1.52E+08	Unlabelled	100	n/a
CDNAPMAE	LysC/GluC	$VEGF_{121}a$	425.66	2	850.31	13.5	1.14E+07	Labelled	1.2	98.8
CDNAPMAE	LysC/GluC	$VEGF_{121}a$	425.66	2	850.31	13.6	9.23E+08	Unlabelled	100	n/a

LS-MS/MS profiles for unlabelled peptide fragments obtained following protease digestion of VEGF or VEGF-TMR. The labeling efficiency of $VEGF_{165}b$-TMR or $VEGF_{121}a$-TMR was determined by comparing the integrated peak areas of the unmodified proteolytic peptides in the labelled and unlabelled samples that were digested with trypsin or LysC/GluC. The analysis indicated 94-99% labelling efficiency. *Mono-isotopic mass at a single charge assuming the peptide takes up a single proton. Mono-isotopic mass [M+H] is calculated as (m/z)*z – (z-1).

Table S2 (relates to Figure 1).

LS-MS/MS profiles for labelled peptide fragments obtained following protease digestion of VEGF or VEGF-TMR.

Peptide sequence	Digest	VEGF isoform	Mass/charge (m/z)	Charge (z)	Mass of labelled peptide [M+H]*	Mass of unlabelled peptide [M+H]*	Mass Difference
CDNAPMAEGGGQNHHEVVK	Trypsin	$VEGF_{165}b$	937.39	3	2810.16	1992.89	817.27
CDNAPMAEGGGQNHHEVVK	Trypsin	$VEGF_{121}a$	937.39	3	2810.16	1992.80	817.27
CDNAPMAE	LysC/GluC	$VEGF_{165}b$	834.30	2	1667.59	850.31	817.28
CDNAPMAE	LysC/GluC	$VEGF_{121}a$	834.30	2	1667.59	850.31	817.28

LS-MS/MS profiles for labelled peptide fragments obtained following protease digestion of VEGF or VEGF-TMR. Observed mass of the labelled peptides containing the N-terminal cysteine confirmed a mass increase of 817 Da due to covalent attachment of the 6-TMR-PEG-CBT. *Mono-isotopic mass at a single charge assuming the peptide takes up a single proton. *Mono-isotopic mass [M+H] is calculated as (m/z)*z – (z-1).

Table S3. Competition binding pK_i values for VEGF-A isoforms binding to VEGFR2 and NRP1 (relates to Figures 3 and 4).

	**NanoLuc-VEGFR2**	**NanoLuc-VEGFR2**	**NanoLuc-VEGFR2**	**NanoLuc-NRP1**
**VEGF isoform**	**$VEGF_{165}$a-TMR**	**$VEGF_{165}$b-TMR**	**$VEGF_{121}$a-TMR**	**$VEGF_{165}$a-TMR**
VEGF-Ax	9.20 ± 0.02	9.57 ± 0.03	9.65 ± 0.06	ND
$VEGF_{165}$a	9.57 ± 0.04	9.73 ± 0.09	9.54 ± 0.05	9.54 ± 0.21
$VEGF_{165}$b	9.07 ± 0.02	9.61 ± 0.06	9.28 ± 0.10	ND
$VEGF_{121}$a	9.30 ± 0.06	9.58 ± 0.18	9.31 ± 0.08	ND
$VEGF_{145}$a	8.83 ± 0.03	8.92 ± 0.08	8.82 ± 0.08	7.82 ± 0.15
$VEGF_{189}$a	9.13 ± 0.03	8.92 ± 0.03	9.06 ± 0.09	8.60 ± 0.14
$VEGF_{111}$a	9.66 ± 0.13	9.89 ± 0.15	9.98 ± 0.05	ND

Summary of binding affinities (pK_i) of unlabelled VEGF-A isoforms determined from inhibition of the binding of $VEGF_{165}$a-TMR, $VEGF_{165}$b-TMR or $VEGF_{121}$a-TMR to NanoLuc-VEGFR2 and NanoLuc-NRP1. Data are expressed as mean ± S.E.M determined from 5 independent experiments. ND = not determined due to lack of significant inhibition of binding with 30nM unlabelled VEGF isoform.
