# Supplementary material for: Real-Time Ligand Binding of Fluorescent VEGF-A Isoforms that Discriminate between VEGFR2 and NRP1 in Living Cells
Source: Cell Chem Biol. 2018 Oct 18;25(10):1208–1218.e5. doi: 10.1016/j.chembiol.2018.06.012 (PMC6200776; doi:10.1016/j.chembiol.2018.06.012)
Supplement: Document S2. Article plus Supplemental Information [file mmc2.pdf]

Article

Cell Chemical Biology

Real-Time Ligand Binding of Fluorescent VEGF-A Isoforms that Discriminate between VEGFR2 and NRP1 in Living Cells

Graphical Abstract

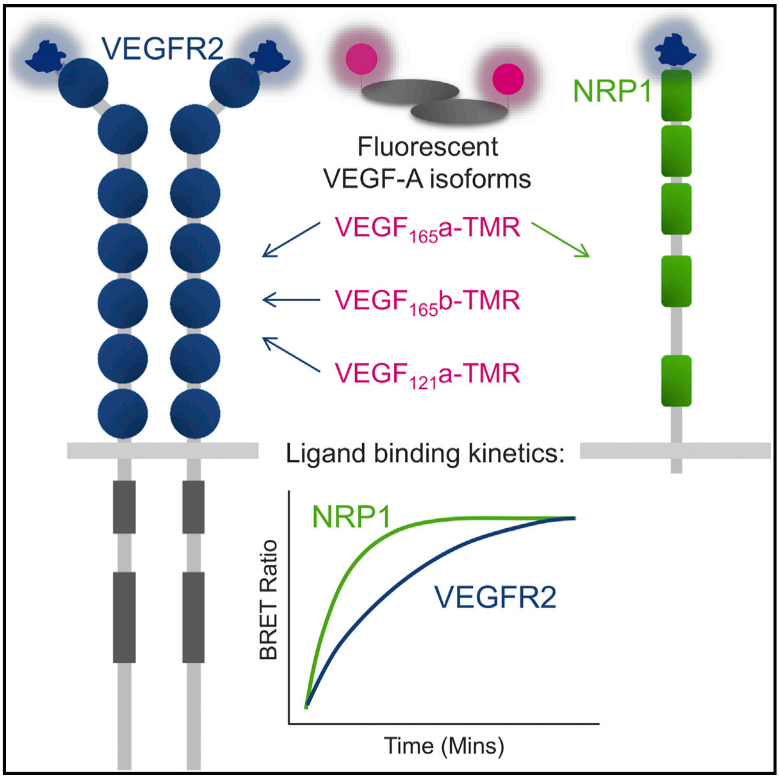

Highlights

- $VEGF_{165}a$, $VEGF_{121}a$, and $VEGF_{165}b$ were single-site labeled with tetramethylrhodamine
- NanoBRET quantified that VEGF-A isoforms have similar binding properties at VEGFR2
- NRP1 expressed in live cells does not bind $VEGF_{165}b$, $VEGF_{121}a$, VEGF-Ax, or $VEGF_{111}a$
- VEGFR2 and NRP1 have markedly distinct kinetic profiles binding $VEGF_{165}a$-TMR

Authors

Chloe J. Peach, Laura E. Kilpatrick, Rachel Friedman-Ohana, ..., Keith V. Wood, Jeanette Woolard, Stephen J. Hill

Correspondence

jeanette.woolard@nottingham.ac.uk (J.W.),
stephen.hill@nottingham.ac.uk (S.J.H.)

In Brief

Peach et al. have used fluorescent VEGF-A isoforms to demonstrate that they can discriminate between VEGFR2 and its co-receptor NRP1 in real-time ligand binding studies in live cells. This precision chemical biology approach showed that fluorescent $VEGF_{165}a$ binds more rapidly to NRP1 than VEGFR2.

Peach et al., 2018, Cell Chemical Biology *25*, 1208–1218
October 18, 2018 © 2018 The Author(s). Published by Elsevier Ltd.
https://doi.org/10.1016/j.chembiol.2018.06.012

CellPress

Real-Time Ligand Binding of Fluorescent VEGF-A Isoforms that Discriminate between VEGFR2 and NRP1 in Living Cells

Chloe J. Peach,[1,2,4] Laura E. Kilpatrick,[1,2,4] Rachel Friedman-Ohana,[3] Kris Zimmerman,[3] Matthew B. Robers,[3] Keith V. Wood,[3] Jeanette Woolard,[1,2,*] and Stephen J. Hill[1,2,5,*]
[1]Division of Physiology, Pharmacology and Neuroscience, School of Life Sciences, University of Nottingham, Nottingham NG7 2UH, UK
[2]Centre of Membrane Proteins and Receptors, University of Birmingham and University of Nottingham, Nottingham NG7 2UH, UK
[3]Promega Corporation, 2800 Woods Hollow Road, Madison, WI 53711, USA
[4]These authors contributed equally
[5]Lead Contact
*Correspondence: jeanette.woolard@nottingham.ac.uk (J.W.), stephen.hill@nottingham.ac.uk (S.J.H.)
https://doi.org/10.1016/j.chembiol.2018.06.012

SUMMARY

Fluorescent VEGF-A isoforms have been evaluated for their ability to discriminate between VEGFR2 and NRP1 in real-time ligand binding studies in live cells using BRET. To enable this, we synthesized single-site (N-terminal cysteine) labeled versions of $VEGF_{165}$a, $VEGF_{165}$b, and $VEGF_{121}$a. These were used in combination with N-terminal NanoLuc-tagged VEGFR2 or NRP1 to evaluate the selectivity of VEGF isoforms for these two membrane proteins. All fluorescent VEGF-A isoforms displayed high affinity for VEGFR2. Only $VEGF_{165}$a-TMR bound to NanoLuc-NRP1 with a similar high affinity (4.4 nM). Competition NRP1 binding experiments yielded a rank order of potency of $VEGF_{165}$a > $VEGF_{189}$a > $VEGF_{145}$a. $VEGF_{165}$b, VEGF-Ax, $VEGF_{121}$a, and $VEGF_{111}$a were unable to bind to NRP1. There were marked differences in the kinetic binding profiles of $VEGF_{165}$a-TMR for NRP1 and VEGFR2. These data emphasize the importance of the kinetic aspects of ligand binding to VEGFR2 and its co-receptors in the dynamics of VEGF signaling.

INTRODUCTION

Angiogenesis, the growth of new blood vessels from pre-existing vasculature, is critical in both physiology and pathology for maintaining an adequate supply of oxygen and nutrients (Chung and Ferrara, 2011). Vascular endothelial growth factor A (VEGF-A) is an essential mediator of both angiogenesis and vascular permeability that signals via its cognate receptor VEGF receptor 2 (VEGFR2) (Koch et al., 2011; Shibuya, 2011). VEGF binds to VEGFR2 at the extracellular immunoglobulin (Ig)-like domains 2 and 3 (D2/D3) of the receptor (Ruch et al., 2007). VEGF binding stimulates receptor dimerization and initiates conformational changes across the VEGFR2 dimer interface that result in auto- and transphosphorylation of intracellular tyrosine residues (Cunningham et al., 1997). Subsequent recruitment of adaptor proteins and activation of downstream signaling cascades leads to cell proliferation, migration, and survival (Koch et al., 2011). VEGFR2 is overexpressed in many solid tumors and leads to activation of pro-angiogenic signaling, which promotes tumorigenesis. As a consequence, a number of anti-angiogenic therapeutics have been targeted at the VEGF/VEGFR2 axis (Ferrara and Adamis, 2016).

VEGFR2 signaling is selectively enhanced by its co-receptor neuropilin-1 (NRP1), a transmembrane glycoprotein that lacks kinase activity and whose upregulation in malignant tumors is correlated to aggressive cancer phenotypes (Jubb et al., 2012; Goel and Mercurio, 2013; Lee et al., 2014). NRP1 is a multifaceted co-receptor that can also bind structurally and functionally unrelated class 3 semaphorins (Djordjevic and Driscoll, 2013; Guo and Vander Kooi, 2015). However, its functional role in vessel development is evident from the severe cardiovascular abnormalities exhibited in *Nrp1* knockout mice (Kitsukawa et al., 1997; Kawasaki et al., 1999; Gu et al., 2003). NRP1 selectively potentiates VEGFR2-mediated endothelial cell motility and vascular permeability without promoting proliferation, driving arterial vessel development *in vivo* (Chittenden et al., 2006; Fantin et al., 2011; Lanahan et al., 2013). While it lacks kinase activity, NRP1 has a short cytoplasmic tail containing a serine-glutamate-alanine motif that interacts with PDZ domain-containing synectin (Cai and Reed, 1999; Wang et al., 2006; Prahst et al., 2008), through which NRP1 may modulate VEGFR2 trafficking or expression (Ballmer-Hofer et al., 2011). VEGF interacts with NRP1 via a C-terminal arginine residue, whereas N-terminal residues on VEGF are responsible for VEGFR2 binding (Djordjevic and Driscoll, 2013; Guo and Vander Kooi, 2015).

VEGF is an anti-parallel disulfide-linked homodimer with multiple endogenous isoforms resulting from alternative mRNA splicing or encoded by separate genes that each elicit different signaling outcomes (Woolard et al., 2009). Alternative splicing of the VEGF-A gene (*Vegfa*) results in isoforms of varying lengths that include the prototypical pro-angiogenic isoform $VEGF_{165}$a and a freely diffusible $VEGF_{121}$a isoform lacking interactions with heparin (Harper and Bates, 2008). Isoforms with a carboxy terminus substituting CDKPRR for SLTRKD, including $VEGF_{165}$b and the more recently identified VEGF-Ax, have reported anti-angiogenic

 © 2018 The Author(s). Published by Elsevier Ltd.
This is an open access article under the CC BY license (http://creativecommons.org/licenses/by/4.0/).

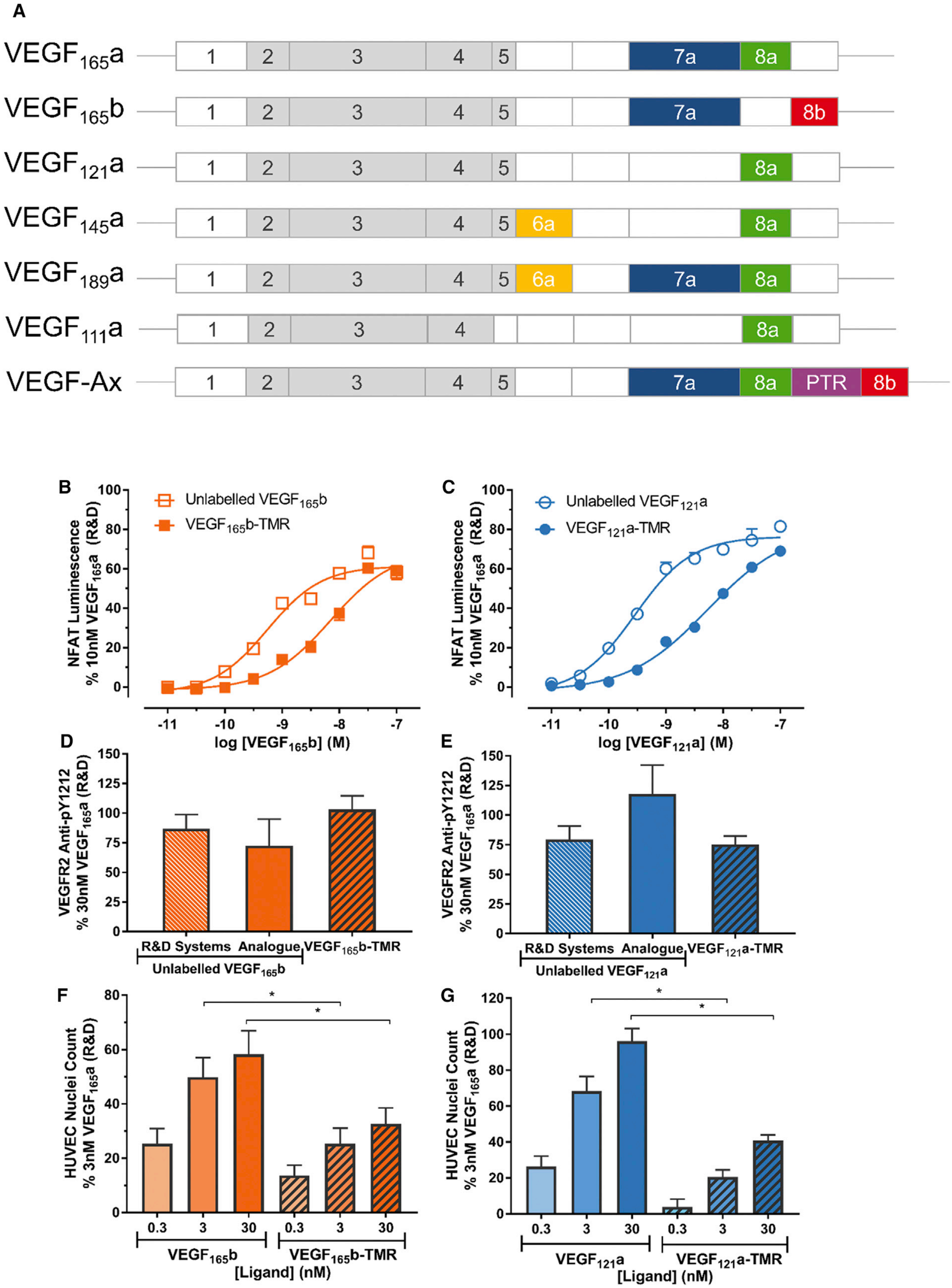

(legend on next page)

activity *in vivo* (Woolard et al., 2004; Cébe Suarez et al., 2006; Eswarappa et al., 2014). Distinct signaling outcomes downstream of VEGFR2 have been suggested to result from different abilities of distinct VEGF isoforms to bind to NRP1 (Simons et al., 2016; Peach et al., 2018). Despite existing anti-cancer therapeutics targeting VEGF and its known modulation by NRP1, there is limited quantitative information on the binding characteristics of specific isoforms at full-length VEGFR2 and NRP1 in living cells.

Significant advances in our understanding of ligand binding to G-protein-coupled receptors (GPCRs), and more recently RTKs, have resulted from the development of fluorescent ligand technologies that use bioluminescence resonance energy transfer (BRET) (Stoddart et al., 2015, 2018). NanoBRET is a proximity-based assay that can quantify interactions between a fluorescent ligand and a receptor fused at its N terminus to a small, bright nanoluciferase (NanoLuc) (Machleidt et al., 2015; Stoddart et al., 2015; Kilpatrick et al., 2017). Having developed a technique to stoichiometrically label $VEGF_{165}a$ with the red-shifted fluorophore tetramethylrhodamine (TMR) (Kilpatrick et al., 2017), we synthesized fluorescent variants of "anti-angiogenic" $VEGF_{165}b$ and freely diffusible $VEGF_{121}a$ to probe their pharmacology at full-length VEGFR2 and its co-receptor NRP1 in living cells at 37°C. We report here the binding affinities and real-time binding kinetics of VEGF-A isoforms to NanoLuc-tagged VEGFR2 and NRP1. We also demonstrate that fluorescent analogs of $VEGF_{165}b$ and $VEGF_{121}a$ can be used to selectively bind to VEGFR2 but not NRP1 in living cells.

RESULTS

Generation and Characterization of Stoichiometrically Labeled $VEGF_{165}b$-TMR and $VEGF_{121}a$-TMR

Synthesis and purification of fluorescent VEGF-A isoforms $VEGF_{165}b$ and $VEGF_{121}a$ (Figure 1A) labeled at a single N-terminal cysteine residue with 6-TMR-PEG-CBT were prepared as described by Kilpatrick et al. (2017). In brief, VEGF isoforms were expressed as secreted N-terminal HaloTag fusions. The linker connecting HaloTag and the VEGF isoforms contained a modified tobacco etch virus recognition site (EDLYFQC), which upon proteolytic cleavage released a VEGF isoform with an N-terminal cysteine residue that can be specifically labeled via 2-cyanobenzothiazole (CBT) condensation.

Labeling specificity of $VEGF_{165}b$-TMR (Figure S1) and $VEGF_{121}a$-TMR (Figure S2) were determined by liquid chromatography-tandem mass spectrometry (LC-MS/MS) analysis of labeled and unlabeled VEGF isoforms that were digested with multiple proteases as described previously for $VEGF_{165}a$-TMR (Kilpatrick et al., 2017). This analysis indicated highly efficient and selective labeling of the N-terminal cysteine residue of each VEGF isoform (Figures S1 and S2). 6-TMR-PEG-CBT chemical modification (817 Da) was identified exclusively on the N-terminal cysteine residue of each VEGF isoform at 97% ($VEGF_{165}b$-TMR) and 94%–99% ($VEGF_{121}a$-TMR) labeling efficiency (Tables S1 and S2). We did not observe non-specific labeling of any of the other cysteine residues present in either $VEGF_{165}b$-TMR or $VEGF_{121}a$-TMR. Fluorescence SDS-PAGE analysis of the purified $VEGF_{165}b$-TMR and $VEGF_{121}a$-TMR isoforms in the presence or absence of 100 mM DTT confirmed that, in non-reducing conditions, both VEGF isoforms were largely present as homodimers (Figures S1 and S2). Deglycosylation by PNGase provided evidence that the purified $VEGF_{165}b$-TMR was glycosylated (Figure S1). However, for $VEGF_{121}a$-TMR, treatment with PNGase strongly suggested that it was present in both glycosylated and non-glycosylated forms under normal conditions (Figure S2). To confirm the concentrations of $VEGF_{165}b$-TMR and $VEGF_{121}a$-TMR (and their dimeric nature), we also undertook fluorescence correlation spectroscopy studies in the presence and absence of 10 mM DTT as described by Kilpatrick et al. (2017) (Figures S1 and S2).

Agonist Activity of Fluorescent VEGF Isoforms in HEK293 Cells and HUVECs

To determine whether the N-terminal TMR labeling of $VEGF_{165}b$ and $VEGF_{121}a$ influenced their VEGFR2 agonist activity, we used a calcium-based nuclear factor of activated T cells (NFAT) reporter gene assay (Carter et al., 2015) to measure signaling downstream of wild-type VEGFR2 expressed in HEK293T cells lacking VEGFR1 or NRP1 (Figure S3). Figure 1 shows the agonist activity of $VEGF_{165}b$-TMR (Figure 1B) and $VEGF_{121}a$-TMR (Figure 1C) compared with the agonist actions of equivalent unlabeled

Figure 1. Functional Characterization of $VEGF_{165}b$-TMR and $VEGF_{121}a$-TMR Activities

(A) Schematic illustrating exons present in different VEGF-A isoforms following alternative mRNA splicing, including the region from post-translational read-through (PTR) in VEGF-Ax.

(B and C) NFAT production in HEK293T cells stably expressing wild-type VEGFR2 in response to 5 hr stimulation with $VEGF_{165}b$-TMR or $VEGF_{165}b$ prepared identically to the fluorescent analog (B), or $VEGF_{121}a$-TMR or unlabeled equivalent $VEGF_{121}a$ (C). Data are mean ± SEM (5 independent experiments, duplicate wells) expressed as a percentage of the response to 10 nM $VEGF_{165}a$ measured in the same experiment.

(D and E) VEGFR2 phosphorylation in HEK293T cells stably expressing NanoLuc-VEGFR2 in response to 20 min stimulation with 30 nM unlabeled $VEGF_{165}b$ (D) or $VEGF_{121}a$ (E). Data are presented for $VEGF_{165}b$ or $VEGF_{121}a$ obtained from a commercial source (R&D Systems) or prepared identically to the TMR analogs (Analogue), or for the fluorescent TMR-labeled variants of each VEGF-A isoform. As a negative control, cells were pre-incubated with 1 μM cediranib for 30 min and stimulated in its presence. Cells were fixed (3% paraformaldehyde [PFA]/PBS), permeabilized (0.025% Triton-X-PBS), blocked for non-specific binding, incubated with an antibody specific for phosphorylated tyrosine 1212, and nuclei stained with H33342. Cells were imaged using an IX Micro widefield platereader (20× objective) and quantified using a granularity algorithm (MetaXpress, Molecular Devices). Data were baseline corrected for non-specific binding (secondary antibody only) and expressed as a percentage normalized to cediranib-treated wells (0%) and response to 30 nM $VEGF_{165}a$ (100%) from 5 independent experiments. Statistical analysis performed using a one-way ANOVA and Sidak's multiple comparisons showed no significance.

(F and G) Comparison of the extent of HUVEC proliferation in response to stimulation with $VEGF_{165}b$ or $VEGF_{165}b$-TMR (F) and $VEGF_{121}a$ or $VEGF_{121}a$-TMR (G) isoforms. Following serum deprivation, HUVECs were stimulated in duplicate wells for 48 hr with 0.3, 3, or 30 nM ligand (37°C/5% CO_2), then fixed using 3% PFA/PBS and nuclei stained with H33342. Cells were imaged using an IX Micro widefield platereader (4× objective) with nuclei counted using a granularity algorithm (MetaXpress, Molecular Devices). Data are expressed as a percentage of the response to 3 nM $VEGF_{165}a$ and represent mean ± SEM from 6 independent experiments. Statistical analyses were performed using a one-way ANOVA and Sidak's multiple comparisons: *$p < 0.05$.

See also Figures S1 and S2; Tables S1 and S2.

VEGF isoforms prepared in a manner identical to that of the fluorescent variant. Each ligand evoked a submaximal response compared with the response obtained with 10 nM $VEGF_{165}a$ (Figures 1B and 1C), consistent with previous work with unlabeled $VEGF_{165}b$ and $VEGF_{121}a$ (Carter et al., 2015; Kilpatrick et al., 2017). However, a comparison of the EC_{50} values of $VEGF_{165}b$-TMR and $VEGF_{121}a$-TMR indicated that the fluorescent ligands had EC_{50} values that were an order of magnitude higher than their unlabeled counterparts ($VEGF_{165}b$-TMR pEC_{50} = 8.16 ± 0.11 versus $VEGF_{165}b$ pEC_{50} = 9.16 ± 0.09; $VEGF_{121}a$-TMR pEC_{50} = 8.57 ± 0.07 versus $VEGF_{121}a$ pEC_{50} = 9.51 ± 0.09; n = 5 in each case). However, in each case the TMR-labeled VEGF isoform produced a maximum response similar to that obtained with the unlabeled $VEGF_{165}b$ or $VEGF_{121}a$ (Figures 1B and 1C). Although untransfected HEK293T cells did show some low-level expression of endogenous VEGFR2 (Figure S3), neither untransfected nor NanoLuc-NRP1-expressing cells produced a measurable NFAT signal in response to $VEGF_{165}a$ (data not shown).

The agonist effect of the two fluorescent ligands was also evaluated for pY1212 phosphorylation of VEGFR2 using a phosphospecific antibody (Figures 1C and 1D). At 30 nM, both ligands were able to stimulate pY1212 phosphorylation to the same extent as the equivalent unlabeled versions of $VEGF_{165}b$ and $VEGF_{121}a$ (Figures 1C and 1D).

Finally, we also investigated agonist activity of these VEGF-A isoforms in human umbilical vein endothelial cells (HUVECs) that endogenously express both VEGFR2 and NRP1 (Figures S3, 1C, and 1D). Immunolabeling of HUVECs showed a minimal presence of endogenous VEGFR1 (Figure S3). Both unlabeled isoforms stimulated a concentration-dependent increase in HUVEC cell proliferation (Figures 1E and 1F). $VEGF_{165}b$ produced a maximum response that was only circa 60% of that obtained with 3 nM $VEGF_{165}a$ (Figure 1E). In contrast, $VEGF_{121}a$ produced a response similar to that obtained with $VEGF_{165}a$ (Figure 1F). Both fluorescent ligands, however, evoked much lower maximal responses (30% for $VEGF_{165}b$-TMR; 40% for $VEGF_{121}a$-TMR) than those obtained with their unlabeled counterparts (Figures 1E and 1F), indicative of partial agonist activity. In keeping with this, the EC_{50} values of the fluorescent isoforms for HUVEC cell proliferation were, however, very similar to the unlabeled $VEGF_{165}b$ and $VEGF_{121}a$ (Figures 1E and 1F). This contrasted markedly with the full agonist response determined previously with $VEGF_{165}a$-TMR in HUVECs (Kilpatrick et al., 2017).

Binding of $VEGF_{165}a$-TMR, $VEGF_{165}b$-TMR, and $VEGF_{121}a$-TMR to VEGFR2

Initial imaging studies were undertaken to monitor the spatial aspects of VEGF isoform binding to HaloTag-labeled VEGFR2 expressed in HEK293T cells (labeled with membrane-impermeant HaloTag-AlexaFluor488 substrate; Figure 2). Under basal conditions, VEGFR2 was located on both the cell membrane and within intracellular sites (indicative of constitutive internalization; Kilpatrick et al., 2017; Figure 2). Following 60-min stimulation with 10 nM $VEGF_{165}a$-TMR (Kilpatrick et al., 2017), $VEGF_{165}b$-TMR, or $VEGF_{121}a$-TMR, there was a clear co-localization with HaloTag-VEGFR2 at both the cell membrane and increased internalized receptor (Figure 2).

NanoBRET was also used to quantify the real-time binding of the three fluorescent VEGF-A isoforms to NanoLuc-tagged VEGFR2 expressed in living HEK293T cells at 37°C. The assay is based on the close proximity (<10 nm) required for bioluminescence energy transfer between the fluorophore of a receptor-bound fluorescent ligand (BRET acceptor) and the N-terminal NanoLuc (BRET donor) of the receptor. Saturable binding of $VEGF_{165}a$-TMR, $VEGF_{165}b$-TMR, and $VEGF_{121}a$-TMR to NanoLuc-VEGFR2 was clearly demonstrated, and this was largely prevented in the presence of 100 nM unlabeled competitor (Figures 3A–3C). Derived equilibrium binding constants revealed that each isoform bound with nanomolar affinity with a rank order $VEGF_{165}a$-TMR > $VEGF_{121}a$-TMR > $VEGF_{165}b$-TMR (Table 1). Real-time binding kinetics measured every 30 s at 37°C showed VEGFR2 binding peaked within 20 min for each VEGF-TMR isoform (Figures 3D–3F). Kinetic binding experiments were conducted with five separate concentrations of VEGF-TMR isoform, which enabled a global fit of the data to provide estimates for k_{on} and k_{off} for each fluorescent ligand. These data showed that $VEGF_{165}a$-TMR had a faster k_{on} than $VEGF_{121}a$-TMR and $VEGF_{165}b$-TMR, but each isoform had similar k_{off} rates (Table 1). The ratio of k_{off}/k_{on} also provided an estimate of the kinetically derived K_D values, which were very similar to those obtained from equilibrium measurements (Table 1).

To gain some insight into whether NanoLuc-VEGFR2 or HaloTag-VEGFR2 were markedly overexpressed in our HEK293T cells, we compared their relative expression levels with those of native untransfected HEK293T and HUVECs using quantitative immunohistochemistry with a selective VEGFR2 antibody (Figure S4). These data showed that the expression levels of the tagged VEGFR2 variants were low and below the native expression level of VEGFR2 in HUVECs (Figure S4).

Using $VEGF_{165}a$-TMR, $VEGF_{165}b$-TMR, and $VEGF_{121}a$-TMR as three distinct fluorescent probes, increasing concentrations of unlabeled VEGF-Ax were used to inhibit the specific binding of each concentration of fluorescent ligand to NanoLuc-VEGFR2 (0.25–3 nM) (Figures 3G–3I). These data were used to derive pK_i values for VEGF-Ax assuming mass action interactions (Table S3). Binding affinities were also derived from similar experiments with a comprehensive panel of unlabeled VEGF-A isoforms at NanoLuc-VEGFR2 (Table S3). pK_i values obtained for each competing ligand were not significantly different between the fluorescent VEGF probes used (one-way ANOVA).

Real-Time Binding of Fluorescent $VEGF_{165}a$ to NRP1

We were also able to apply the NanoBRET technology to the type I single transmembrane co-receptor NRP1. NanoLuc was fused to the extracellular N terminus of NRP1 and expressed in HEK293T cells to isolate binding of the different fluorescent VEGF-A isoforms to full-length NRP1. Specific binding of $VEGF_{165}a$-TMR to NanoLuc-NRP1 was clearly observed with minimal non-specific binding following incubation for 60 min (K_D = 4.41 ± 1.34 nM, n = 5; Figure 4A). Kinetic binding measurements also revealed that specific binding of $VEGF_{165}a$-TMR to NanoLuc-NRP1 was reached within 4 min and exhibited faster k_{on} (7.11 ± 2.33 × 10^7 min^{-1} M^{-1}) and k_{off} (0.26 ± 0.05 min^{-1}) rate constants than were achieved with this ligand at NanoLuc-VEGFR2 (Figure 4B and Table 1). However, the equilibrium dissociation constants were very similar for $VEGF_{165}a$-TMR between NRP1 and VEGFR2 (Table 1). Displacing each concentration of $VEGF_{165}a$-TMR (0.5–5 nM) by increasing concentrations

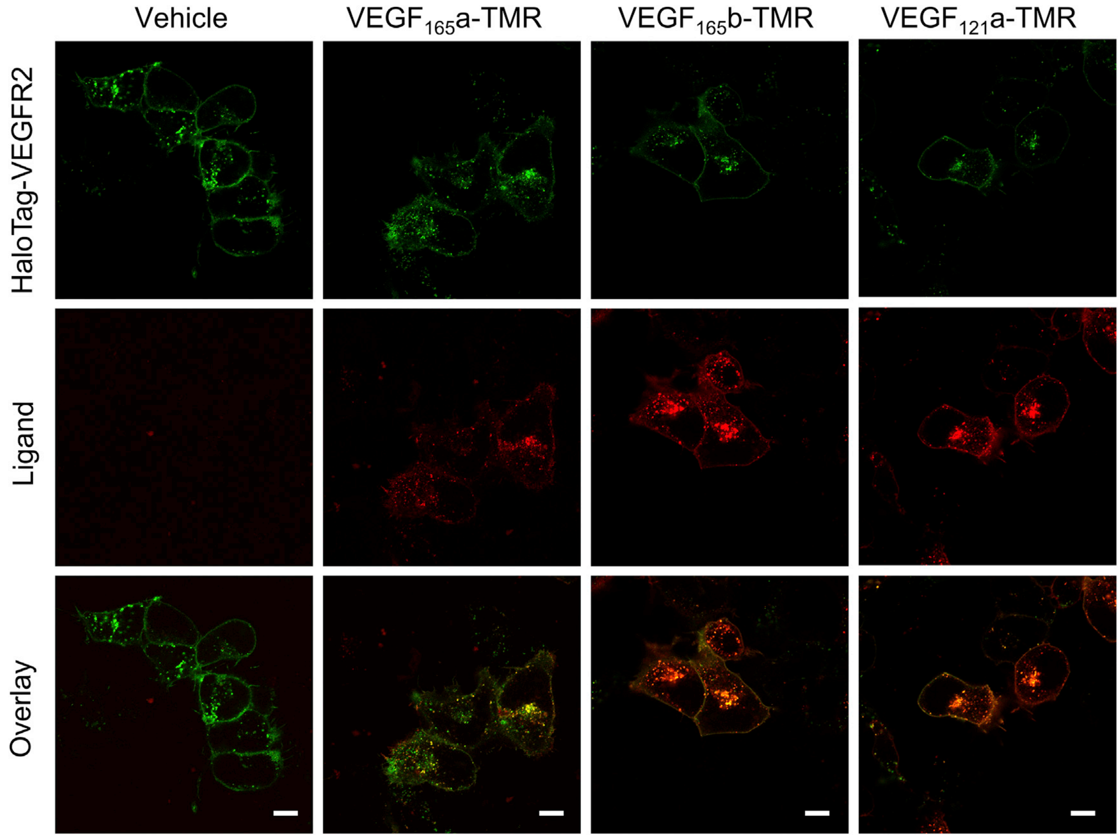

Figure 2. Co-localization of Fluorescent VEGF-A Isoform Binding and HaloTag-VEGFR2

Confocal images of HEK293T cells stably expressing HaloTag-VEGFR2 (green) stimulated with vehicle or 10 nM $VEGF_{165}$a-TMR, $VEGF_{165}$b-TMR, or $VEGF_{121}$a-TMR (red) for 1 hr at 37°C. Cells were imaged live using a Zeiss LSM710 and are representative images of 3 independent experiments. Scale bar, 10 μm. See also Figures S3 and S4.

of unlabeled $VEGF_{165}$a showed competitive inhibition, yielding a pK_i of 9.54 ± 0.21 (Figure 4C, n = 5; Table S3). A linear relationship was observed between the IC_{50} and $VEGF_{165}$a-TMR concentration at NanoLuc-NRP1 (R^2 = 0.95, p < 0.005; Figure 4D).

NRP1 Expressed in Living Cells Does Not Bind $VEGF_{165}$b, $VEGF_{121}$a, VEGF-Ax, or $VEGF_{111}$a

To investigate how the three distinct fluorescent VEGF isoforms interacted with NRP1, we used $VEGF_{165}$a-TMR alongside $VEGF_{165}$b-TMR and $VEGF_{121}$a-TMR to image fluorescent ligand binding to HaloTag-NRP1 expressed in HEK293T cells and labeled with membrane-impermeant Alexa Fluor 488. Upon both vehicle and fluorescent ligand application, HaloTag-NRP1 remained at the cell surface (Figure 5A). While 10 nM $VEGF_{165}$a-TMR co-localized with HaloTag-NRP1 when imaged after 60 min, no binding of $VEGF_{165}$b-TMR and $VEGF_{121}$a-TMR to HaloTag-NRP1 was detected (Figure 5A). This latter observation was confirmed using NanoBRET, whereby no saturable binding was detected between $VEGF_{165}$b-TMR or $VEGF_{121}$a-TMR and NanoLuc-NRP1 (Figure 5B). Using 3 nM $VEGF_{165}$a-TMR as a fluorescent probe, only unlabeled $VEGF_{165}$a, $VEGF_{145}$a, and $VEGF_{189}$a displaced binding from NRP1 (Figure 5C). Full competition ligand binding experiments allowed pK_i values at NanoLuc-NRP1 to be determined for these latter VEGF-A isoforms (Table S3). Quantitative immunohistochemistry analysis confirmed that NanoLuc-NRP1 and HaloTag-NRP1 were expressed at low levels in HEK293T cells (Figure S4).

NanoBRET was also used to investigate ligand binding at a previously identified VEGF binding-dead mutant NRP1 Y297A, lacking a key residue in the b1 domain responsible for VEGF binding (Fantin et al., 2014). Having also confirmed membrane expression of HaloTag-NRP1 Y297A using live cell imaging, co-localization was absent for all three fluorescent VEGF isoforms (Figure 6A). Analogous BRET experiments showed that $VEGF_{165}$a-TMR did not interact with NanoLuc-NRP1 Y297A, yielding BRET ratios that did not differ from vehicle (Figure 6B). This confirmed NRP1 Y297A as a mutant deficient for VEGF binding.

DISCUSSION

In the present study we have evaluated the ability of three fluorescent analogs of VEGF ($VEGF_{165}$a, $VEGF_{165}$b, and $VEGF_{121}$a) to discriminate between VEGFR2 and NRP1 in living cells in real time. To enable this, we prepared single-site (N-terminal cysteine) labeled versions of $VEGF_{165}$b and $VEGF_{121}$a essentially as described previously for $VEGF_{165}$a (Kilpatrick et al., 2017). These fluorescent ligands were used in combination with

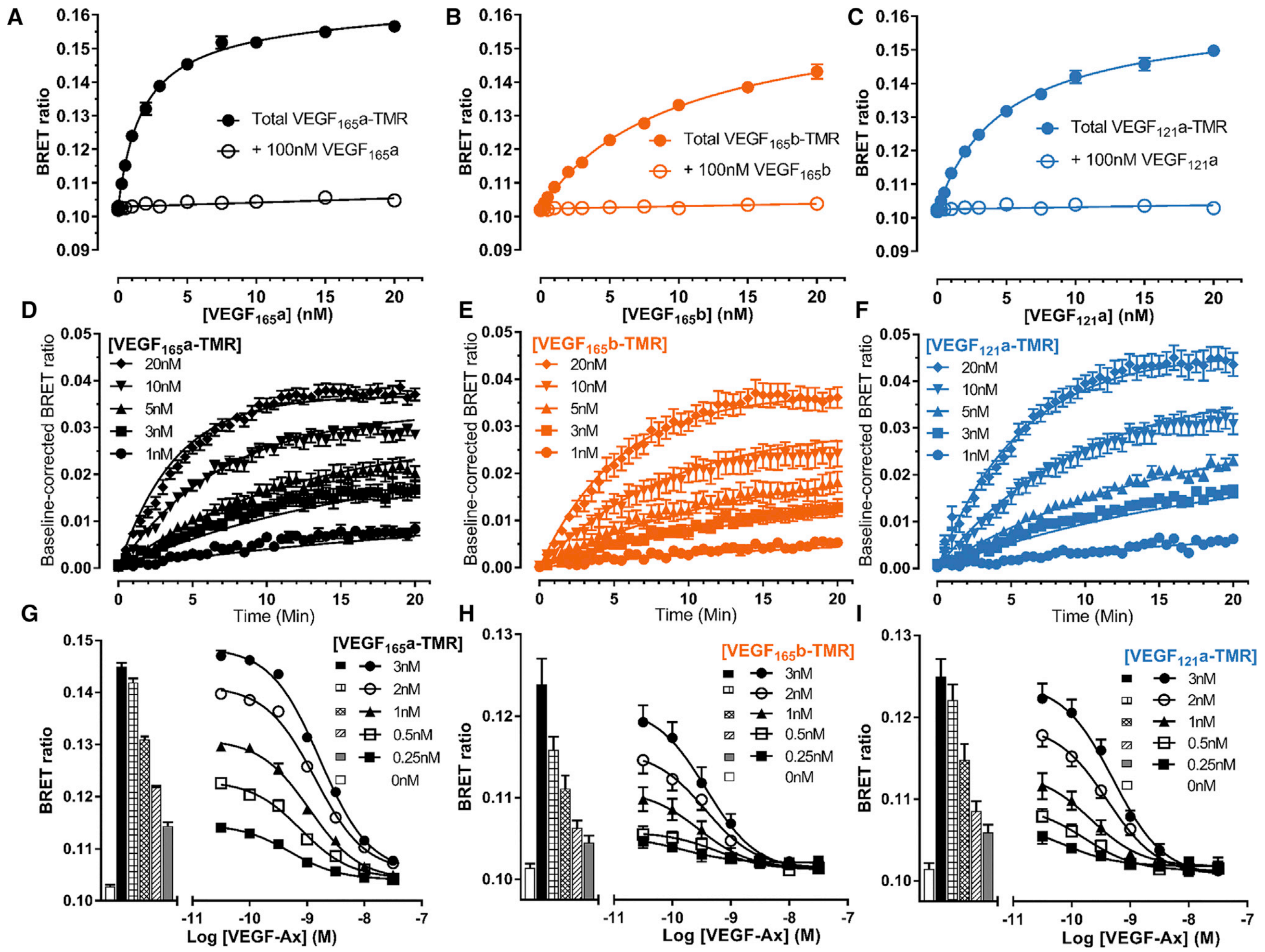

Figure 3. Binding Characteristics of Fluorescent VEGF Isoforms to NanoLuc-VEGFR2 Expressed in HEK293 Cells

(A–C) HEK293T cells expressing N-terminal NanoLuc-VEGFR2 were incubated with increasing concentrations of (A) $VEGF_{165}$a-TMR, (B) $VEGF_{165}$b-TMR, or (C) $VEGF_{121}$a-TMR, in the presence and absence of 100 nM unlabeled VEGF, added simultaneously to define non-specific binding (60 min; 37°C). BRET ratios are expressed as mean ± SEM from 5 independent experiments with duplicate wells. Where not shown, error bars are within the size of the symbol.

(D–F) Time course of (D) $VEGF_{165}$a-TMR, (E) $VEGF_{165}$b-TMR, or (F) $VEGF_{121}$a-TMR ligand binding kinetics at NanoLuc-VEGFR2. Cells treated with furimazine were left to equilibrate for 5 min before addition of 1–20 nM fluorescent VEGF ligand or vehicle, and measurements were taken every 30 s for 20 min (37°C). Baseline BRET ratios are corrected to vehicle at time zero. Data represent mean ± SEM from 5 independent experiments, and individual curves were fitted with a simple exponential association model.

(G–I) Displacement of (G) $VEGF_{165}$a-TMR, (H) $VEGF_{165}$b-TMR, or (I) $VEGF_{121}$a-TMR binding by unlabeled VEGF-Ax. Increasing concentrations of VEGF-Ax were added in duplicate wells simultaneously with 5 separate fixed concentrations (0.25–3 nM) of $VEGF_{165}$a-TMR, $VEGF_{165}$b-TMR, or $VEGF_{121}$a-TMR (60 min, 37°C). Raw BRET ratios from 5 independent experiments are shown as mean ± SEM with bars illustrating vehicle (white bars) or fluorescent VEGF-TMR alone.

HEK293T cells stably expressing N-terminal NanoLuc-tagged VEGFR2 or NRP1 to evaluate the selectivity of VEGF isoforms for these two membrane proteins. The close proximity requirements (<10 nm) of the interaction between fluorescent ligand and receptor protein in order for bioluminescence transfer to occur (for NanoBRET measurement) ensured a high specificity of interaction, regardless of the extent of endogenous receptor expression. This was important since, although HEK293T did not express endogenous NRP1 (Figure S3), endogenous VEGFR2 were detected in a subpopulation of untransfected HEK293T cells. Furthermore, following expression of HaloTag-labeled NRP1, the endogenous expression of VEGFR2 appeared to increase (Figure S3). The expression level of VEGFR1 was, however, minimal in both untransfected HEK293T cells and those transfected with tagged variants of either VEGFR2 or NRP1.

$VEGF_{165}$a-TMR, $VEGF_{165}$b-TMR, and $VEGF_{121}$a-TMR each exhibited saturable binding to NanoLuc-VEGFR2 expressed in HEK293T cells with nanomolar affinity. Furthermore, there were minimal levels of non-specific binding detected with each fluorescent ligand. Analysis of the real-time binding characteristics of each fluorescent ligand indicated that all three fluorescent VEGF variants had very similar k_{on} and k_{off} rate constants, and indeed their off rates were very slow (k_{off} = 0.05–0.06 min^{-1}).

Table 1. Binding Characteristics of Fluorescent Ligands Binding to VEGFR2 or NRP1

Fluorescent Ligand	Receptor	Saturation K_D (nM)	Kinetic K_D (nM)	k_{on} (min^{-1} M^{-1})	k_{off} (min^{-1})
$VEGF_{165}$a-TMR	NanoLuc-VEGFR2	2.03 ± 0.51	6.64 ± 4.37	$1.54 \times 10^7 \pm 0.38 \times 10^7$	0.06 ± 0.02
$VEGF_{165}$b-TMR	NanoLuc-VEGFR2	9.53 ± 1.36	11.3 ± 3.54	$7.29 \times 10^6 \pm 1.84 \times 10^6$	0.06 ± 0.01
$VEGF_{121}$a-TMR	NanoLuc-VEGFR2	5.54 ± 1.34	5.75 ± 0.46	$8.51 \times 10^6 \pm 0.81 \times 10^6$	0.05 ± 0.00
$VEGF_{165}$a-TMR	NanoLuc-NRP1	4.41 ± 1.34	4.95 ± 1.25	$7.11 \times 10^7 \pm 2.33 \times 10^7$	0.26 ± 0.05

Equilibrium binding parameters for fluorescent VEGF isoforms derived from saturation and kinetic NanoBRET experiments, showing equilibrium dissociation (K_D), association rate (k_{on}), and dissociation rate (k_{off}) constants at NanoLuc-VEGFR2 and NanoLuc-NRP1. Data are expressed as mean ± SEM determined from 5 independent experiments.

pK_i values were obtained for a panel of seven unlabeled VEGF-A isoforms, including the recently described VEGF-Ax (Eswarappa et al., 2014), from competition experiments using all three of the fluorescent probes. All seven ligands had comparable nanomolar binding affinities for VEGFR2 ranging between 0.2 and 1.4 nM, in agreement with previous studies (Peach et al., 2018), suggesting that potential differences in signaling responses of these isoforms is not due to binding alone (Whitaker et al., 2001; Cébe Suarez et al., 2006; Eswarappa et al., 2014; Kilpatrick et al., 2017). There was no evidence of probe dependence in the measurement of these equilibrium constants, suggesting that the interactions could be described by simple mass action interactions.

$VEGF_{165}$a-TMR bound to NanoLuc-NRP1 in living cells with a high affinity (4.41 nM) similar to that observed at NanoLuc-VEGFR2 (2.03 nM). However, in marked contrast $VEGF_{165}$b-TMR and $VEGF_{121}$a-TMR did not bind to NanoLuc-NRP1 (measured via NanoBRET) at concentrations up to 20 nM. This observation was corroborated by live cell confocal imaging, which showed that $VEGF_{165}$b-TMR (10 nM) and $VEGF_{121}$a-TMR (10 nM) bound to HaloTag-VEGFR2 but not to HaloTag-NRP1. The importance of residue Y297 (Fantin et al., 2014) of NRP1 for the binding of $VEGF_{165}$a was confirmed in HEK293T cells expressing a Y297A mutant of NRP1. Competition binding experiments at NanoLuc-NRP1 yielded a rank order of pK_i values of $VEGF_{165}$a > $VEGF_{189}$a > $VEGF_{145}$a. In contrast, $VEGF_{165}$b, VEGF-Ax, $VEGF_{121}$a, and $VEGF_{111}$a were unable to displace 3 nM $VEGF_{165}$a-TMR at concentrations up to 30 nM. These observations support previous reports that these isoforms may be unable to bind NRP1 (Woolard et al., 2009). There have, however, been conflicting reports regarding $VEGF_{121}$a binding to NRP1 (reviewed in Sarabipour and Mac Gabhann, 2017). Thus, although radioligand binding and solid-phase biotinylation assays have shown no interaction between $VEGF_{121}$a and NRP1 (Cébe Suarez et al., 2006; Kawamura et al., 2008; Xin et al., 2016), low-affinity binding was detected using immobilized monomeric NRP1 and surface plasmon resonance (SPR) or isolated NRP1 b1/b2 domains (Pan et al., 2007; Parker et al., 2012; Delcombel et al., 2013).

A key feature of the present study is the ability to study the binding of VEGF-A isoforms to full-length VEGFR2 and NRP1 in living cells and in real time. This ensures that the interactions studied are of physiological relevance (Djordjevic and Driscoll, 2013). The lack of binding of $VEGF_{165}$b, VEGF-Ax, $VEGF_{121}$a, and $VEGF_{111}$a to NRP1 is seen at concentrations up to 20 nM, which are far in excess of the predicted

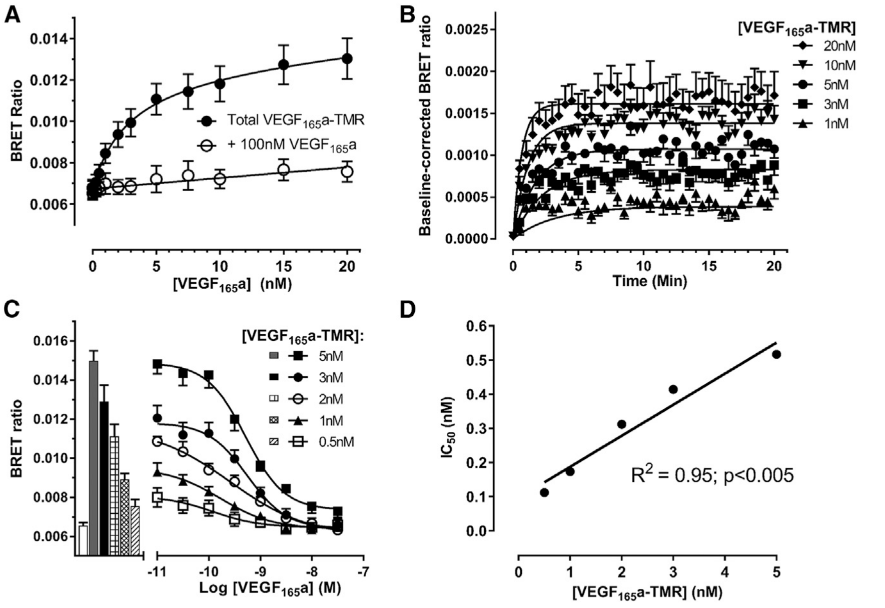

Figure 4. Binding Characteristics of $VEGF_{165}$a Binding to NanoLuc-NRP1

(A) Increasing concentrations of $VEGF_{165}$a-TMR were added to HEK293T cells stably expressing N-terminal NanoLuc-NRP1 in the presence and absence of 100 nM unlabeled $VEGF_{165}$a to determine non-specific binding, and cells were incubated for 60 min at 37°C. Raw BRET ratios are expressed as mean ± SEM from 5 independent experiments.

(B) Time course of $VEGF_{165}$a-TMR binding to NanoLuc-NRP1. BRET ratios were baseline corrected to vehicle, curves were fitted to a simple exponential association model, and data are shown as mean ± SEM from 5 independent experiments.

(C) Inhibition of the binding of $VEGF_{165}$a-TMR (0.5, 1, 2, 3, and 5 nM) to NanoLuc-NRP1 by increasing concentrations of unlabeled $VEGF_{165}$a added simultaneously and incubated for 60 min at 37°C. Raw BRET ratios from 5 independent displacement experiments using duplicate wells are shown as mean ± SEM with bars representing vehicle (white) or $VEGF_{165}$a-TMR only.

(D) Linear regression analysis (R^2 = 0.95; $p < 0.005$) of the relationship between IC_{50} values determined in (C) and $VEGF_{165}$a-TMR concentration. The y intercept provides an estimate for the K_i of competing $VEGF_{165}$a (0.10 nM), while the slope (0.09) represents the ratio K_i/K_D thus yielding an estimated K_D = 1.11 nM for $VEGF_{165}$a-TMR at NanoLuc-NRP1.

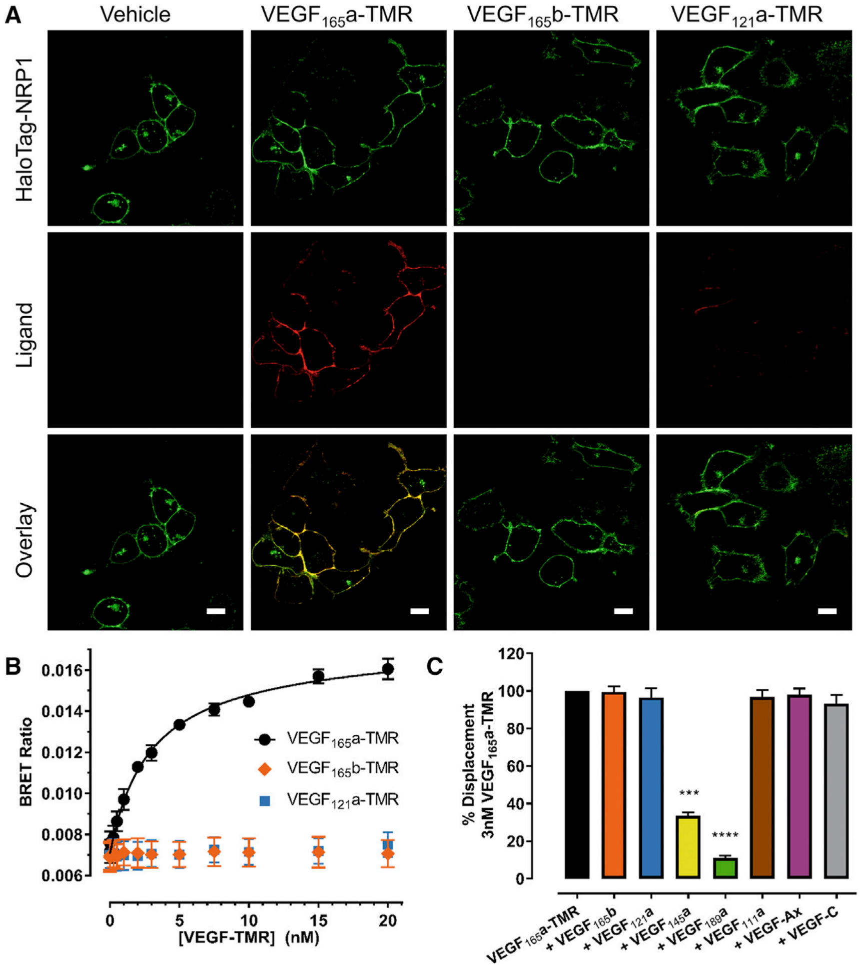

Figure 5. Selective Binding of VEGF Isoforms at NRP1

(A) Confocal live cell imaging of fluorescently labeled VEGF-TMR isoforms binding to N-terminal HaloTag-NRP1 stably expressed in HEK293T cells. HaloTag-NRP1 was tagged with the membrane-impermeant HaloTag-AF488 dye (green) and then incubated with 10 nM $VEGF_{165}$a-TMR, $VEGF_{165}$b-TMR, or $VEGF_{121}$a-TMR (red) for 60 min at 37°C. Cells were imaged using an LSM710 confocal microscope and images are representative of those obtained in 3 independent experiments. Scale bar, 10 μm.

(B) NanoLuc-NRP1 HEK293T cells were incubated with increasing concentrations of $VEGF_{165}$a-TMR, $VEGF_{165}$b-TMR, or $VEGF_{121}$a-TMR and incubated for 60 min at 37°C. Raw BRET ratios are expressed as mean ± SEM from 3–4 independent experiments.

(C) Inhibition of $VEGF_{165}$a-TMR (3 nM) by competing unlabeled VEGF isoforms (30 nM), added simultaneously and incubated for 60 min at 37°C. Data are normalized to 3 nM $VEGF_{165}$a-TMR (100%, black bar) and represent mean ± SEM pooled from 5 independent experiments. Statistical analyses were performed using Welch's t test: ***$p \leq 0.001$; ****$p \leq 0.0001$.

physiological levels of these ligands (<1 nM; Clegg and Mac Gabhann, 2017). These data suggest that $VEGF_{165}$b-TMR and $VEGF_{121}$a-TMR can be used as selective fluorescent probes for VEGFR2, even in cells that also express endogenous NRP1.

Real-time analysis of the binding of $VEGF_{165}$a-TMR to NanoLuc-NRP1 expressed in HEK293T cells enabled the kinetics of ligand binding to be monitored to these membrane proteins for the first time. Despite comparable equilibrium dissociation constants determined by saturation and kinetic binding experiments, $VEGF_{165}$a-TMR had faster binding kinetics at NRP1 compared with VEGFR2. Maximum specific binding to NanoLuc-NRP1 could be achieved within 5 min largely as a consequence of its very fast k_{off} (0.26 min^{-1}). These data suggest that in cells expressing both VEGFR2 and NRP1, $VEGF_{165}$a will bind more quickly to NRP1 than to VEGFR2, particularly at low agonist concentrations. This may have important implications for the dynamics of VEGF signaling, and emphasize the need to understand the kinetic aspects of ligand binding to VEGFR2 and its co-receptors as well as the temporal aspects of intracellular signaling. Thus, since

$$t_{1/2} = \frac{0.693}{k_{on}\ x\ [A] + k_{off}}$$

for 1 nM $VEGF_{165}$a-TMR using the parameters provided in Table 1, the $t_{1/2}$ for association to VEGFR2 will be 9.2 min while that for NRP1 will be 2.1 min. For 10 nM $VEGF_{165}$a-TMR the $t_{1/2}$ values are 3.2 min and 0.7 min for VEGFR2 and NRP1, respectively.

Imaging ligand/receptor interactions using a membrane-impermeant HaloTag label also highlighted distinct differences in the subcellular distributions of VEGFR2 and NRP1, and the consequences of incubation with $VEGF_{165}$a. HaloTag-VEGFR2 was constitutively internalized in the absence of ligand stimulation. This agrees with previous antibody-based imaging in HUVECs and human microvascular endothelial cells (Gampel et al., 2006; Basagiannis and Christoforidis, 2016; Basagiannis et al., 2016), and our own studies using VEGFR2 stably expressed in HEK293T cells (Kilpatrick et al., 2017). Furthermore, $VEGF_{165}$a-TMR, $VEGF_{165}$b, and $VEGF_{121}$a were able to stimulate VEGFR2 internalization. In contrast, HaloTag-NRP1, labeled with a cell-impermeant HaloTag dye, was largely expressed on the cell membrane of HEK293T cells and remained at the cell surface despite 60 min of stimulation with a high concentration of $VEGF_{165}$a-TMR. Furthermore, $VEGF_{165}$a-TMR only labeled membrane-expressed NRP1. Other groups have shown an intracellular NRP1 distribution using permeabilized fluorescent antibody labeling (Narazaki and Tosato, 2006; Ballmer-Hofer et al., 2011). However, it is clear from the present work that cell membrane NRP1 is the primary target for $VEGF_{165}$a and that this VEGF-A isoform does not stimulate internalization of NRP1.

It has been previously noted that fluorescent ligands can have pharmacological properties very different from their unlabeled counterparts and that they should be evaluated as new chemical

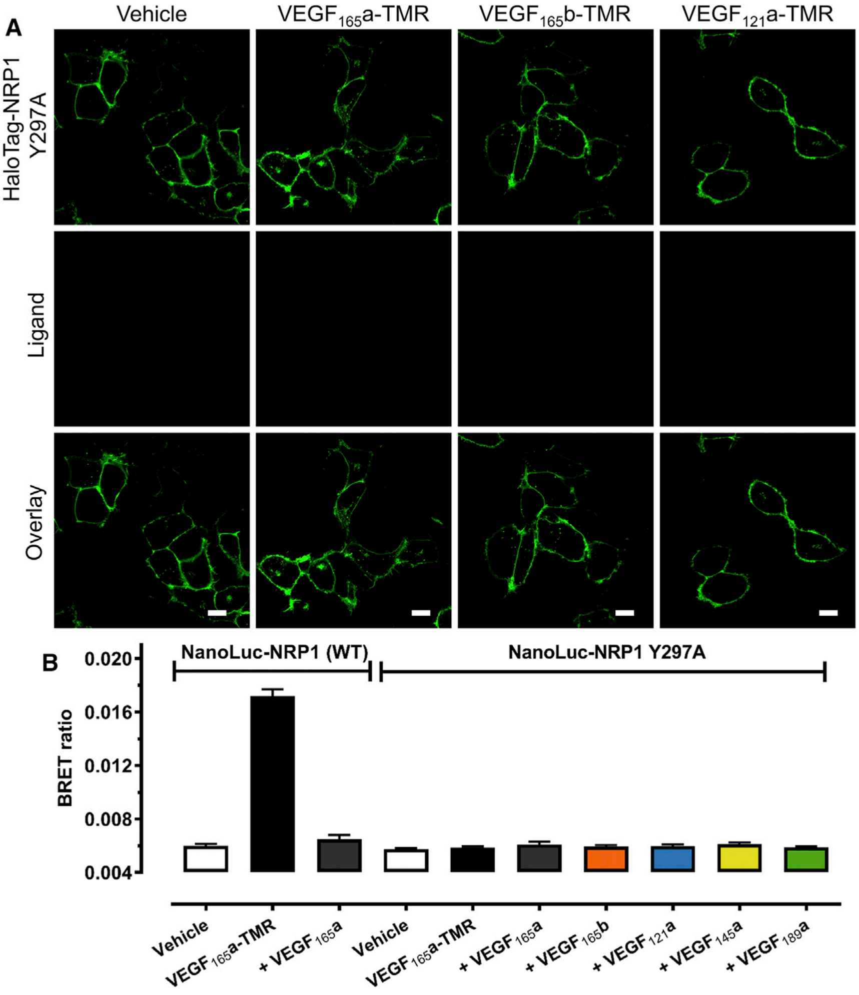

Figure 6. The NRP1 Mutant Y297A Is Unable to Bind Any VEGF Isoforms

(A) Live confocal imaging of HEK293T cells stably expressing mutant HaloTag-NRP1 Y297A (green) labeled with membrane-impermeant HaloTag-AF488 dye (green). Cells were stimulated with 10 nM $VEGF_{165}$a-TMR, $VEGF_{165}$b-TMR, or $VEGF_{121}$a-TMR for 60 min at 37°C. Cells were imaged using an LSM710 confocal microscope, and images are representative images of 3 independent experiments. Scale bar, 10 μm.

(B) NanoBRET measurements of the effect of unlabeled VEGF isoforms (30 nM) on the binding of 3 nM $VEGF_{165}$a-TMR to wild-type NanoLuc-NRP1 or NanoLuc-NRP1 Y297A stably expressing HEK293T for 60 min (37°C). Raw BRET ratios are expressed as mean ± SEM pooled from 4 independent experiments.

entities (Stoddart et al., 2015, 2016). We have previously shown that $VEGF_{165}$a-TMR behaves very similarly to $VEGF_{165}$a in its ability to (1) stimulate NFAT reporter gene responses in HEK293T cells expressing wild-type VEGFR2 and (2) enable proliferation of HUVECs (Kilpatrick et al., 2017). However, both $VEGF_{165}$b-TMR and $VEGF_{121}$a-TMR behave differently in functional assays to $VEGF_{165}$b and $VEGF_{121}$a prepared in an identical way to the fluorescent probes. Thus, in NFAT assays the EC_{50} values obtained with both $VEGF_{165}$b-TMR (pEC_{50} = 8.28) and $VEGF_{121}$a-TMR (pEC_{50} = 8.57) were an order of magnitude higher (less potent) than the non-fluorescent versions. However, these EC_{50} values were very similar to the pK_D values obtained from saturation binding studies (7.9–8.1 for $VEGF_{165}$b-TMR and 8.2–8.4 for $VEGF_{121}$a-TMR) and from competition binding studies (9.29–9.30 for $VEGF_{165}$b and 9.16–9.59 for $VEGF_{121}$a). This suggests that the differences were predominantly affinity based and that there was little signal amplification in the NFAT assay. Comparison of the agonist effects of fluorescent $VEGF_{165}$b and $VEGF_{121}$a on pY1212 phosphorylation, however, indicated that they produced the same maximal response as their unlabeled counterparts. In the HUVEC proliferation assay both $VEGF_{165}$b-TMR and $VEGF_{121}$a-TMR appeared to be of lower efficacy than the non-fluorescent ligands but still showed partial agonism in stimulating HUVEC proliferation. Taken together, these data suggest that $VEGF_{165}$b-TMR and $VEGF_{121}$a-TMR, unlike $VEGF_{165}$a-TMR, are lower-affinity and lower-efficacy agonists at VEGFR2 than their unlabeled analogs. Furthermore, the extent of agonist activity appears to depend on the signaling pathway being monitored. This may indicate an ability for these fluorescent analogs to exhibit some signaling bias in a similar way to that seen with G-protein-coupled receptors (Smith et al., 2018).

In summary, fluorescent VEGF isoforms were used to probe the pharmacology of VEGFR2 and its co-receptor NRP1 in living cells in real time at 37°C. Despite approved therapeutics targeting VEGF/VEGFR2 (Ferrara and Adamis, 2016), this is the first comprehensive ligand binding study of the interactions of a range of VEGF isoforms with both full-length VEGFR2 and NRP in living cells. The real-time sensitivity of NanoBRET revealed clear differences in the kinetic binding profiles of $VEGF_{165}$a-TMR for NRP1 and VEGFR2, despite this ligand having a very similar equilibrium dissociation binding constant for each membrane protein. All VEGF isoforms studied had a similar high affinity for VEGFR2 but not all isoforms interacted with NRP1. In particular, $VEGF_{165}$b-TMR and $VEGF_{121}$a-TMR were not able to bind to NRP1 at physiologically relevant concentrations. These two partial agonist ligands should therefore be important and selective probes for the study of VEGFR2 in cells also expressing NRP1. Furthermore, our study also emphasizes the importance of the kinetic aspects of ligand binding to VEGFR2 and its co-receptors in the overall dynamics of VEGF signaling.

SIGNIFICANCE

VEGF-A is an essential mediator of angiogenesis that signals via VEGFR2. We have synthesized fluorescent VEGF-A isoforms and demonstrate that they can discriminate

between VEGFR2 and its co-receptor NRP1 in real-time ligand binding studies. We have used a precision chemical biology approach in live cells to accurately define the binding characteristics of specific VEGF-A isoforms and to determine which isoforms can bind to NRP1 at concentrations required to occupy VEGFR2. Only $VEGF_{165}$a, $VEGF_{145}$a, and $VEGF_{189}$a are able to also bind to NRP1. Furthermore, we have shown that while $VEGF_{165}$a-TMR has a similar equilibrium binding affinity for VEGFR2 and NRP1, it binds more rapidly to NRP1 than to VEGFR2. We have also shown that $VEGF_{165}$a-TMR has a shorter residence time ($1/k_{off}$) at NRP1 (3.8 min) than VEGFR2 (16.6 min). These fluorescent ligands should therefore serve as valuable probes to interrogate the roles of VEGFR2 and NRP1 in angiogenesis and signaling.

STAR★METHODS

Detailed methods are provided in the online version of this paper and include the following:

- KEY RESOURCES TABLE
- CONTACT FOR REAGENT AND RESOURCE SHARING
- EXPERIMENTAL MODEL AND SUBJECT DETAILS
- METHOD DETAILS
  - Cell Culture
  - DNA Constructs
  - Fluorescent Ligand Synthesis
  - NFAT Luciferase Reporter Gene Assay
  - VEGFR2 Phosphorylation Assay
  - HUVEC Proliferation Assay
  - Measuring Ligand Binding Using NanoBRET
  - Live Cell Confocal Imaging
  - Fluorescence Correlation Spectroscopy (FCS)
  - Immunofluorescence Labelling
- QUANTIFICATION AND STATISTICAL ANALYSIS
  - Data Analysis
  - High Content Imaging
  - FCS Autocorrelation Analysis
- DATA AND SOFTWARE AVAILABILITY

SUPPLEMENTAL INFORMATION

Supplemental Information includes four figures and three tables and can be found with this article online at https://doi.org/10.1016/j.chembiol.2018.06.012.

ACKNOWLEDGMENTS

This work was supported by the Biotechnology and Biological Sciences Research Council (grant number BB/L019418/1) and Promega Corporation. C.P. was funded by an A.J. Clark studentship from the British Pharmacological Society (BPS). We thank Dave Good and Sergy Levin for the synthesis of 6-TMR-PEG-CBT and Mike Rosenblatt for the LC-MS/MS analysis. We also thank the School of Life Sciences Imaging (SLIM) team for maintenance and support for high content and confocal imaging facilities.

AUTHOR CONTRIBUTIONS

Conceptualization, S.J.H., J.W., and L.E.K.; Methodology, S.J.H., L.E.K., R.F.-O., and K.Z.; Formal analysis, C.J.P., L.E.K., S.J.H. and R.F.-O.; Investigation, C.J.P., L.E.K., R.F.-O., M.B.R., S.J.H., and J.W.; Writing – Original Draft, C.J.P., L.E.K., and S.J.H.; Writing – Review and Editing, C.J.P., L.E.K., K.V.W., M.B.R., R.F.-O, J.W., and S.J.H; Supervision, S.J.H., J.W., and L.E.K.

DECLARATION OF INTERESTS

R.F.-O., M.B.R., K.Z., and K.V.W. are employees of Promega Corporation, which has proprietary rights over the NanoBRET assay, HaloTag technology, and CBT labeling technology.

Received: February 2, 2018
Revised: May 23, 2018
Accepted: June 29, 2018
Published: July 26, 2018

REFERENCES

Ballmer-Hofer, K., Andersson, A.E., Ratcliffe, L.E., and Berger, P. (2011). Neuropilin-1 promotes VEGFR-2 trafficking through Rab11 vesicles thereby specifying signal output. Blood *118*, 816–826.

Basagiannis, D., and Christoforidis, S. (2016). Constitutive endocytosis of VEGFR2 protects the receptor against shedding. J. Biol. Chem. *291*, 16892–16903.

Basagiannis, D., Zografou, S., Murphy, C., Fotsis, T., Morbidelli, L., Ziche, M., Bleck, C., Mercer, J., and Christoforidis, S. (2016). VEGF induces signalling and angiogenesis by directing VEGFR2 internalisation via macropinocytosis. J. Cell Sci. *129*, 4091–4104.

Cai, H., and Reed, R.R. (1999). Cloning and characterization of neuropilin-1-interacting protein: a PSD- 95/Dlg/ZO-1 domain-containing protein that interacts with the cytoplasmic domain of neuropilin-1. J. Neurosci. *19*, 6519–6527.

Carter, J., Wheal, A., Hill, S., and Woolard, J. (2015). Effects of receptor tyrosine kinase inhibitors on $VEGF_{165}$a- and $VEGF_{165}$b-stimulated gene transcription in HEK-293 cells expressing human VEGFR2. Br. J. Pharmacol. *172*, 3141–3150.

Cébe Suarez, S., Pieren, M., Cariolato, L., Arn, S., Hoffman, U., Bogucki, A., Manlius, C., Wood, J., and Ballmer-Hofer, K. (2006). A VEGF-A splice variant defective for heparan sulfate and neuropilin-1 binding shows attenuated signaling through VEGFR-2. Cell. Mol. Life Sci. *63*, 2067–2077.

Chittenden, T.W., Claes, F., Lanahan, A.A., Autiero, M., Palac, R.T., Tkachenko, E.V., Elfenbein, A., Ruiz de Almodovar, C., Dedkov, E., Tomanek, R., et al. (2006). Selective regulation of arterial branching morphogenesis by synectin. Dev. Cell *10*, 783–795.

Chung, A.S., and Ferrara, N. (2011). Developmental and pathological angiogenesis. Annu. Rev. Cell Dev. Biol. *27*, 563–584.

Clegg, L.E., and Mac Gabhann, F. (2017). A computational analysis of in vivo VEGFR activation by multiple co-expressed ligands. PLoS Comput. Biol. *13*, e1005445.

Cunningham, S.A., Arrate, M.P., Brock, T.A., and Waxham, M.N. (1997). Interactions of FLT-1 and KDR with phospholipase $C\gamma$: identification of the phosphotyrosine binding sites. Biochem. Biophys. Res. Commun. *240*, 635–639.

Delcombel, R., Janssen, L., Vassy, R., Gammons, M., Haddad, O., Richard, B., Letourneur, D., Bates, D., Hendricks, C., Waltenberger, J., et al. (2013). New prospects in the roles of the C-terminal domains of VEGF-A and their cooperation for ligand binding, cellular signaling and vessels formation. Angiogenesis *16*, 353–371.

Djordjevic, S., and Driscoll, P.C. (2013). Targeting VEGF signalling via the neuropilin co-receptor. Drug Discov. Today *18*, 447–455.

Eswarappa, S.M., Potdar, A.A., Koch, W.J., Fan, Y., Vasu, K., Lindner, D., Willard, B., Graham, L.M., Dicorleto, P.E., and Fox, P.L. (2014). Programmed translational readthrough generates antiangiogenic VEGF-Ax. Cell *157*, 1605–1618.

Fantin, A., Schwarz, Q., Davidson, K., Normando, E.M., Denti, L., and Ruhrberg, C. (2011). The cytoplasmic domain of neuropilin 1 is dispensable for angiogenesis, but promotes the spatial separation of retinal arteries and veins. Development *138*, 4185–4191.

Fantin, A., Herzog, B., Mahmoud, M., Yamaji, M., Plein, A., Denti, L., Ruhrberg, C., and Zachary, I. (2014). Neuropilin 1 (NRP1) hypomorphism combined with defective VEGF-A binding reveals novel roles for NRP1 in developmental and pathological angiogenesis. Development *141*, 556–562.

Ferrara, N., and Adamis, A.P. (2016). Ten years of anti-vascular endothelial growth factor therapy. Nat. Rev. Drug Discov. *15*, 385–403.

Gampel, A., Moss, L., Jones, M.C., Brunton, V., Norman, J.C., and Mellor, H. (2006). VEGF regulates the mobilization of VEGFR2/KDR from an intracellular endothelial storage compartment. Blood *108*, 2624–2631.

Goel, H.L., and Mercurio, A.M. (2013). VEGF targets the tumour cell. Nat. Rev. Cancer *13*, 871–882.

Gu, C., Rodriguez, E.R., Reimert, D.V., Shu, T., Fritzsch, B., Richards, L.J., Kolodkin, A.L., and Ginty, D.D. (2003). Neuropilin-1 conveys semaphorin and VEGF signaling during neural and cardiovascular development. Dev. Cell *5*, 45–57.

Guo, H.F., and Vander Kooi, C.W. (2015). Neuropilin functions as an essential cell surface receptor. J. Biol. Chem. *290*, 29120–29126.

Harper, S.J., and Bates, D. (2008). VEGF-A splicing: the key to anti-angiogenic therapeutics? Nat. Rev. Cancer *8*, 880–887.

Jubb, A.M., Strickland, L.A., Liu, S.D., Mak, J., Schmidt, M., and Koeppen, H. (2012). Neuropilin-1 expression in cancer and development. J. Pathol. *226*, 50–60.

Kawamura, H., Li, X., Harper, S., Bates, D., and Claesson-Welsh, L. (2008). Vascular endothelial growth factor (VEGF)-A_{165}b is a weak in vitro agonist for VEGF receptor-2 due to lack of coreceptor binding and deficient regulation of kinase activity. Cancer Res. *68*, 4683–4692.

Kawasaki, T., Kitsukawa, T., Bekku, Y., Matsuda, Y., Sanbo, M., Yagi, T., and Fujisawa, H. (1999). A requirement for neuropilin-1 in embryonic vessel formation. Development *126*, 4895–4902.

Kilpatrick, L.E., Friedman-Ohana, R., Alcobia, D.C., Riching, K., Peach, C.J., Wheal, A.J., Briddon, S.J., Robers, M.B., Zimmerman, K., Machleidt, T., et al. (2017). Real-time analysis of the binding of fluorescent VEGF165a to VEGFR2 in living cells: effect of receptor tyrosine kinase inhibitors and fate of internalized agonist-receptor complexes. Biochem. Pharmacol. *136*, 62–75.

Kitsukawa, T., Shimizu, M., Sanbo, M., Hirata, T., Taniguchi, M., Bekku, Y., Yagi, T., and Fujisawa, H. (1997). Neuropilin–semaphorin III/D-mediated chemorepulsive signals play a crucial role in peripheral nerve projection in mice. Neuron *19*, 995–1005.

Koch, S., Tugues, S., Li, X., Gualandi, L., and Claesson-Welsh, L. (2011). Signal transduction by vascular endothelial growth factor receptors. Biochem. J. *437*, 169–183.

Lanahan, A., Zhang, X., Fantin, A., Zhuang, Z., Rivera-Molina, F., Speichinger, K., Prahst, C., Zhang, J., Wang, Y., Davis, G., et al. (2013). The neuropilin 1 cytoplasmic domain is required for VEGF-A-dependent arteriogenesis. Dev. Cell *25*, 156–168.

Lee, S.W., Lee, J.E., Yoo, C.Y., Ko, M.S., Park, C.S., and Yang, S.H. (2014). NRP-1 expression is strongly associated with the progression of pituitary adenomas. Oncol. Rep. *32*, 1537–1542.

Machleidt, T., Woodroofe, C.C., Schwinn, M.K., Méndez, J., Robers, M., Zimmerman, K., Otto, P., Daniels, D.L., Kirkland, T.A., and Wood, K.B. (2015). NanoBRET—a novel BRET platform for the analysis of protein-protein interactions. ACS Chem. Biol. *10*, 1797–1804.

Narazaki, M., and Tosato, G. (2006). Ligand-induced internalization selects use of common receptor neuropilin-1 by VEGF165 and semaphorin3A. Blood *107*, 3892–3901.

Pan, Q., Chathery, Y., Wu, Y., Rathore, N., Tong, R., Peale, F., Bagri, A., Tessier-Lavigne, M., Koch, A.W., and Watts, R.J. (2007). Neuropilin-1 binds to VEGF121 and regulates endothelial cell migration and sprouting. J. Biol. Chem. *282*, 24049–24056.

Parker, M.W., Xu, P., Guo, H.F., and Vander Kooi, C.W. (2012). Mechanism of selective VEGF-a binding by neuropilin-1 reveals a basis for specific ligand inhibition. PLoS One *7*, e49177.

Peach, C.J., Mignone, V.W., Augusta Arruda, M., Alcobia, D.C., Hill, S.J., Kilpatrick, L.E., and Woolard, J. (2018). Molecular pharmacology of VEGF-A isoforms: binding and signalling at VEGFR2. Int. J. Mol. Sci. *19*, 1264.

Prahst, C., Héroult, M., Lanahan, A.A., Uziel, N., Kessler, O., Shraga-Heled, N., Simons, M., Neufeld, G., and Augustin, H.G. (2008). Neuropilin-1-VEGFR-2 complexing requires the PDZ-binding domain of neuropilin-1. J. Biol. Chem. *283*, 25110–25114.

Ruch, C., Skiniotis, G., Steinmetz, M.O., Walz, T., and Ballmer-Hofer, K. (2007). Structure of a VEGF–VEGF receptor complex determined by electron microscopy. Nat. Struct. Mol. Biol. *14*, 249–250.

Sarabipour, S., and Mac Gabhann, F. (2017). VEGF-A121a binding to neuropilins—a concept revisited. Cell Adh. Migr. https://doi.org/10.1080/19336918.2017.1372878.

Shibuya, M. (2011). Vascular endothelial growth factor (VEGF) and its receptor (VEGFR) signaling in angiogenesis: a crucial target for anti- and pro-angiogenic therapies. Genes Cancer *2*, 1097–1105.

Simons, M., Gordon, E., and Claesson-Welsh, L. (2016). Mechanisms and regulation of endothelial VEGF receptor signalling. Nat. Rev. Mol. Cell Biol. *17*, 611–625.

Smith, J.S., Lefkowitz, R.J., and Rajagopal, S. (2018). Biased signalling: from simple switches to allosteric microprocessors. Nat. Rev. Drug Discov. *17*, 243–269.

Stoddart, L.A., Johnstone, E.K.M., Wheal, A.J., Goulding, J., Robers, M.B., Machleidt, T., Wood, K.V., Hill, S.J., and Pfleger, K.D.G. (2015). Application of BRET to monitor ligand binding to GPCRs. Nat. Methods *12*, 661–663.

Stoddart, L.A., White, C.W., Nguyen, K., Hill, S.J., and Pfleger, K.D. (2016). Fluorescence- and bioluminescence-based approaches to study GPCR ligand binding. Br. J. Pharmacol. *173*, 3028–3037.

Stoddart, L.A., Kilpatrick, L.E., and Hill, S.J. (2018). NanoBRET approaches to study ligand binding to GPCRs and RTKs. Trends Pharmacol. Sci. *39*, 136–147.

Wang, L., Mukhopadhyay, D., and Xu, X. (2006). C terminus of RGS-GAIP-interacting protein conveys neuropilin-1-mediated signaling during angiogenesis. FASEB J. *20*, 1513–1515.

Whitaker, G.B., Limberg, B.J., and Rosenbaum, J.S. (2001). Vascular endothelial growth factor receptor-2 and neuropilin-1 form a receptor complex that is responsible for the differential signaling potency of $VEGF_{165}$ and $VEGF_{121}$. J. Biol. Chem. *276*, 25520–25531.

Woolard, J., Wang, W., Bevan, H.S., Qiu, Y., Morbidelli, L., Pritchard-Jones, R.O., Cui, T., Sugiono, M., Waine, E., Perrin, R., et al. (2004). $VEGF_{165}$b, an inhibitory vascular endothelial growth factor splice variant: mechanism of action, in vivo effect on angiogenesis and endogenous protein expression. Cancer Res. *64*, 7822–7835.

Woolard, J., Bevan, H.S., Harper, S.J., and Bates, D. (2009). Molecular diversity of VEGF-A as a regulator of its biological activity. Microcirculation *16*, 572–592.

Xin, H., Zhong, C., Nudleman, E., and Ferrara, N. (2016). Evidence for pro-angiogenic functions of VEGF-Ax. Cell *167*, 275–284.

STAR★METHODS

KEY RESOURCES TABLE

REAGENT or RESOURCE	SOURCE	IDENTIFIER
Antibodies		
Mouse monoclonal anti-VEGFR1	Sigma Aldrich	Cat# V4762 RRID:AB_477622
Mouse monoclonal anti-VEGFR2	Sigma Aldrich	Cat# V9134 RRID:AB_477630
Goat polyclonal anti-Neuropilin-1	Santa Cruz	Cat# SC7239 RRID:AB_2150835
Rabbit monoclonal anti-VEGFR2 phosphoY1212	Cell Signalling Technology	Cat# 2477S RRID:AB_331374
Chemicals, Peptides, and Recombinant Proteins		
$VEGF_{165}a$	R&D Systems (Abingdon, UK)	Cat# 293-VE
$VEGF_{165}b$	R&D Systems (Abingdon, UK)	Cat# 3045-VE
$VEGF_{121}a$	R&D Systems (Abingdon, UK)	Cat# 4644-VS
$VEGF_{145}a$	R&D Systems (Abingdon, UK)	Cat# 7626-VE
$VEGF_{189}a$	R&D Systems (Abingdon, UK)	Cat# 8147-VE
$VEGF_{111}a$	R&D Systems (Abingdon, UK)	Cat# 5336-VE
VEGF-Ax	R&D Systems (Abingdon, UK)	Cat# 9018-VE
HaloTag AlexaFluor 488 membrane impermeant substrate	Promega Corporation (Wisconsin, USA)	Cat# G1002
bisBenzimide H 33342 trihydrochloride	Sigma Aldrich	Cat# B2261
Formaldehyde solution 4%	Sigma Aldrich	Cat# F8775
Cediranib	Sequoia Research Products	Cat# SRP01883c
Chromasolv	Sigma Aldrich	Cat# 34877
Rhodamine 6G	Sigma Aldrich	Cat# R4127
Triton-X-100 (laboratory grade)	Sigma Aldrich	Cat# X100
DTT 1,4-Dithiothreitol	Sigma Aldrich	Cat# DTT-RO
PNGase F	Promega Corporation (Wisconsin, USA)	Cat# V4831
Protease-free bovine serum albumin	Milpore	Cat# 126609
Protease-free bovine serum albumin	Sigma Aldrich	Cat# 03117332001
Secondary chick anti-mouse	Invitrogen	Cat# A21463
Secondary donkey anti-goat	Invitrogen	Cat# A11056
Secondary chick anti-rabbit AlexaFluor-488	ThermoFisher Scientific, USA	Cat# A-21441
Chicken serum	Sigma Aldrich	Cat# C5405
Donkey serum	Sigma Aldrich	Cat# D9663
ProLong Gold antifade reagent	ThermoFisher Scientific, USA	Cat# P10144
Dulbecco's Modified Eagle's Medium	Sigma Aldrich	Cat# D6429
Fetal Bovine Serum	Sigma Aldrich	Cat# F2442
Medium 200 (Gibco)	ThermoFisher Scientific, USA	Cat# M-200-500
Large Vessel Endothelial Supplement (LVES 50x) (Gibco)	ThermoFisher Scientific, USA	Cat# A1460801
Poly-D-Lysine hydrobromide	Sigma Aldrich	Cat# P6407
Dulbecco's Phosphate Buffered Saline (DPBS)	Sigma Aldrich	Cat# D8537
Trypsin-EDTA solution x10	Sigma Aldrich	Cat# T4174
Critical Commercial Assays		
HaloTag Mammalian Protein Detection and Purification System	Promega Corporation (Wisconsin, USA)	Cat# G6795
ONE-Glo™ Luciferase	Promega Corporation (Wisconsin, USA)	Cat# E6120
Nano-Glo luciferase assay system (Furimazine)	Promega Corporation (Wisconsin, USA)	Cat# N1130

(*Continued on next page*)

Continued		
REAGENT or RESOURCE	SOURCE	IDENTIFIER
Experimental Models: Cell Lines		
Human: GloResponse™ NFAT-RE-*luc2P* HEK293 cell line (female)	Promega Corporation (Wisconsin, USA)	Cat# E8510
Human: HUVEC cells (newborn male, single donor)	ThermoFisher Scientific	Cat# C0035C. Lot number: 1606186.
Human: HEK293T cells (female)	ATCC (Virginia, USA)	Cat# CRL-3216
Recombinant DNA		
NanoLuc-VEGFR2	Promega Corporation (Wisconsin, USA)	Custom synthesis
NanoLuc-NRP1	Promega Corporation (Wisconsin, USA)	Custom synthesis
NanoLuc-NRP1 Y297A	Promega Corporation (Wisconsin, USA)	Custom synthesis
HaloTag-VEGFR2	Promega Corporation (Wisconsin, USA)	Custom synthesis
HaloTag-NRP1	Promega Corporation (Wisconsin, USA)	Custom synthesis
HaloTag-NRP1 Y297A	Promega Corporation (Wisconsin, USA)	Custom synthesis
$VEGF_{165}a$	Gene Dynamics LLC (Oregon, USA)	Custom synthesis
$VEGF_{165}b$	Gene Dynamics LLC (Oregon, USA)	Custom synthesis
$VEGF_{121}a$	Gene Dynamics LLC (Oregon, USA)	Custom synthesis
pFN21 HaloTag CMV Flexi Vector (modified to contain a IL-6 secretion sequence and a EPTTEDLYFQCDN linker sequence)	Promega Corporation (Wisconsin, USA)	Cat# G2821
Software and Algorithms		
GraphPad Prism 7.02	GraphPad Software, La Jolla California USA	www.graphpad.com
Zen 2010	Zeiss, Germany	www.zeiss.com
MetaXpress	Molecular Devices, USA	www.moleculardevices.com
Other		
Black 96-well plates	Greiner Bio-One	Cat# 655090
White 96-well plates	Greiner Bio-One	Cat# 655098
8-well plates	Nunc Lab-Tek, Thermo Fisher Scientific	Cat# 155411
Coverslips (18x18mm; 1.5H)	Zeiss, Germany	Cat# 474030-9000-000

CONTACT FOR REAGENT AND RESOURCE SHARING

Further information and requests for resources and reagents should be directed to and will be fulfilled by the Lead Contact, Stephen J. Hill (stephen.hill@nottingham.ac.uk).

EXPERIMENTAL MODEL AND SUBJECT DETAILS

HUVECs (obtained from a single newborn male donor) and HEK293T (female) cells were transfected and cultured as described in Method Details.

METHOD DETAILS

Cell Culture

HEK293T cells were grown in Dulbecco's Modified Eagle's Medium (DMEM; Sigma-Aldrich, USA) supplemented with 10% Fetal Calf Serum (FCS; Sigma-Aldrich, USA) at 37°C/5% CO_2. Cells were passaged at 70-80% confluency using Phosphate Buffered Saline (PBS; Lonza, Switzerland) and trypsin (0.25% w/v in versene; Lonza). Stable and transient transfections were performed using FuGENE HD (Promega Corporation, USA) at a reagent to cDNA ratio of 3:1. Human umbilical vein endothelial cells (HUVECs; C0035C, Thermo Fisher Scientific, USA) were grown at 37°C/5% CO_2 in Medium 200 containing 10% Large Vessel Endothelial Supplement (LVES, 50X; Thermo Fisher Scientific, USA) and passaged at 80-90% confluency between passages 4 to 9.

DNA Constructs

For N terminal NanoLuc tagged wildtype VEGFR2 (NM_002253; Genscript, New Jersey, USA) or NRP1 constructs (NM_003873.5; Kazusa DNA Research Institute (Japan) the appropriate cDNA was cloned into a pF-sNnK CMV/neo vector (Promega Corporation;

N1321) encoding the secretory signal peptide sequence of IL-6 fused onto the N terminus of NanoLuc. This resulted in open reading frames which encoded a secreted NanoLuc fused via a Gly-Ser-Ser-Gly (AIA) linker to the N terminus of wildtype VEGFR2 or NRP1 (termed NanoLuc VEGFR2 or NRP1 respectively). For N terminal HaloTag constructs, wildtype VEGFR2 or NRP1 cDNA was cloned into a pFN21A CMV/neo flexi vector (Promega Corporation; G2821) encoding a fusion of the secretory signal peptide sequence of IL-6 onto the N terminus of HaloTag. The resultant ORFs encoded a secreted HaloTag fused via a EPTTEDLYFQSDN(AIA) linker to the N terminus of NRP1 (HaloTag VEGFR2 or NRP1).

Fluorescent Ligand Synthesis

VEGF-A isoforms $VEGF_{165}$a, $VEGF_{165}$b and $VEGF_{121}$a labelled at a single N-terminal cysteine residue with 6- tetramethylrhodamine (TMR)-PEG-CBT were synthesised and purified using the HaloTag mammalian protein detection and purification system (G6795; Promega Corporation, USA) alongside unlabelled analogues prepared identically (as described in Kilpatrick et al., 2017). To generate labelled isoforms, the HaloTEV proteolytic release was done in the presence of 100μM TCEP and 4x molar excess of 6-TMR-PEG-CBT. This step generated VEGF isoforms with an N-terminal cysteine that served as single point of conjugation with 6-TMR-PEG-CBT. The purified labelled isoforms were dialyzed for 24 hours (50mM HEPES, 150mM NaCl) to remove the unconjugated 6-TMR-PEG-CBT and TCEP and stored in 2.5mg/ml protease-free bovine serum albumin (BSA; Millipore, USA) at -80°C. Labelling specificity and efficiency was determined using liquid chromatography-tandem mass spectrometry (LC-MS). SDS-PAGE assays in the presence and absence of 100mM dithiothreitol (DTT; Sigma-Aldrich, UK) or PNGase (Promega Corporation, USA) were used to measure dimerisation and glycosylation status respectively (detailed in Kilpatrick et al., 2017). Ligands were stored in 2.5mg/ml protease-free bovine serum albumin (BSA; Millipore, USA). Labelling specificity and efficiency was determined using liquid chromatography-tandem mass spectrometry (LC-MS). SDS-PAGE assays in the presence and absence of dithiothreitol (DTT; Sigma-Aldrich, UK) or PNGase (Promega Corporation, USA) were used to measure dimerisation and glycosylation status respectively (detailed in Kilpatrick et al., 2017).

NFAT Luciferase Reporter Gene Assay

HEK293T cells stably expressing both wild type VEGFR2 and the Firefly luciferase reporter gene ReLuc2P (Promega Corporation, USA) inserted downstream of the NFAT promoter were used to monitor NFAT-induced gene transcription following VEGFR2 activation (Carter et al., 2015). On the day of experimentation, cells grown to 95-100% confluency were plated in white-sided 96 well plates (Greiner Bio-One, 655089) at 44,000 cells/well, and incubated for 1 hour in 100μl/well serum free DMEM/0.1% BSA (37°C/5% CO_2). Cells were stimulated in duplicate wells with increasing concentrations of $VEGF_{121}$a-TMR, $VEGF_{165}$b-TMR or equivalent unlabelled VEGF isoforms (synthesised in an identical manner to the fluorescent variant), then incubated for 5 hours at 37°C/5% CO_2. ONE-Glo Luciferase reagent (Promega Corporation, USA) was then added at 100μl/well and luminescence was measured using a TopCount platereader (Perkin Elmer, UK) following a 5 minute delay allowing reagent to react with luciferase and background luminescence to subside.

VEGFR2 Phosphorylation Assay

HEK293T cells stably expressing NanoLuc-VEGFR2 were seeded at 15,000 cells/well in black flat-bottomed 96-well plates (Greiner Bio-One, 655090) pre-coated with poly-D-lysine (0.01mg/ml in PBS). Following 24 hours, cells were serum starved and grown for another 24 hours (37°C/5% CO_2), with additional 1 hour serum starving step prior to experimentation. For negative control wells, cells were pre-incubated for 30 minutes with 1μM cediranib (Sequoia Research Products, UK). Cells were then stimulated for 20 minutes with 30nM $VEGF_{165}$b-TMR or $VEGF_{121}$a-TMR, commercially available $VEGF_{165}$a, $VEGF_{165}$b or $VEGF_{121}$a (R&D Systems) and $VEGF_{165}$b or $VEGF_{121}$a prepared identically to the fluorescent analogues, in the presence of absence of negative control 1μM cediranib. Cells were washed with 100μl/well PBS, fixed with 3% paraformaldehyde (PFA)/PBS for 20min at room temperature (RT), washed (3x5min PBS), permeabilised with 0.025% Triton-X-100 in PBS, washed (3x5min PBS) and incubated with 3% BSA/1% glycine/PBS to reduce non-specific binding (30mins, RT). After washing (3x5min PBS), cells were blocked with 10% chick serum in PBS (30min, RT) and incubated at 4°C overnight with rabbit monoclonal anti-VEGFR2 phosphoY1212 (Cell Signalling, 2477) diluted 1:200 in 10% chick serum/PBS. Cells were washed (3x5min PBS) and incubated in the dark with secondary antibody chick anti-rabbit AlexaFluor488 (Thermo Fisher, A21441). Nuclei were stained with 2mg/ml H33342 (15min, RT), washed and stored at 4°C in PBS. Cells were imaged using an ImageXpress Micro widefield platereader (Molecular Devices, USA) with a 20x objective at 4 sites per well using FITC and DAPI filters (exposure 1500ms and 25ms respectively).

HUVEC Proliferation Assay

HUVECs (passage 4-9) were seeded at 5,000 cells/well in black flat-bottomed 96-well plates (Greiner Bio-One, 655090) in 10% LVES/Medium 200. Following 24 hours of cell growth at 37°C/5% CO_2, plating medium was replaced with Medium 200 containing 0.1% serum for 24 hours. Cells were then stimulated with commercially available $VEGF_{121}$a or $VEGF_{165}$b (R&D Systems), $VEGF_{121}$a-TMR or $VEGF_{165}$b-TMR (Promega Corporation, USA) at 0.3nM, 3nM or 30nM (in 0.1% serum/medium), or positive control 3nM $VEGF_{165}$a (R&D Systems). Following 48 hour stimulation at 37°C/5% CO_2, cells were washed with 100μl/well PBS, fixed with 3% PFA/PBS (20 minutes, room temperature) and nuclei stained with 2mg/ml H33342 (15 minutes, RT). Nuclei were imaged using an ImageXpress Micro widefield platereader (Molecular Devices, USA) with a 4x objective using a DAPI filter (4 sites per well, 25ms exposure time).

Measuring Ligand Binding Using NanoBRET

HEKT293 cells stably expressing full-length wild-type VEGFR2, NRP1 or NRP1 Y297A, tagged on the N-terminus with the 19kDa luciferase NanoLuc, were seeded 24 hours prior to experimentation at 35,000 cells/well on white 96-well clear bottomed plates (Greiner Bio-One, 655089) pre-coated with poly-D-lysine (0.01mg/ml in PBS), and incubated at 37°C/5% CO_2. Having identified a natural polymorphism (V297I) in the NanoLuc-VEGFR2 construct used previously (Kilpatrick et al., 2017), experiments performed with $VEGF_{165}$a-TMR verified no distinction from wild type VEGFR2 (Figures 1 and 3). Medium was replaced with Hank's buffered saline solution (HBSS) containing 0.1% BSA. For full displacement experiments, cells were co-incubated with increasing concentrations of unlabelled ligand (R&D Systems) or vehicle (HBSS/0.1% BSA), as well as fixed concentrations of fluorescently labelled $VEGF_{165}$a-TMR, $VEGF_{165}$b-TMR or $VEGF_{121}$a-TMR in duplicate wells (0.25nM, 0.5nM, 1nM, 2nM, 3nM). Additional displacement experiments incubated NanoLuc-VEGFR2 or NanoLuc-NRP1 cells with 3nM VEGF-TMR in the presence and absence of 30nM competing unlabelled VEGF. For saturation experiments, increasing concentrations of $VEGF_{165}$a–TMR, $VEGF_{165}$b-TMR or $VEGF_{121}$a-TMR were added in the presence or absence of a high concentration of corresponding unlabelled ligand (100nM, ~100-fold greater than the estimated K_D value). Following 60min stimulation in the dark at 37°C, the NanoLuc substrate furimazine (final concentration 10μM) was added to each well and equilibrated for 5 minutes to enable NanoLuc-mediated furimazine oxidation and resulting bioluminescence emission. Emissions were recorded using the PHERAstar FS platereader (BMG Labtech) using filters measuring NanoLuc emissions at 450nm (30nm bandpass), then TMR emissions using a longpass filter at 550nm for NanoLuc-VEGFR2 cells or 610nm for cells expressing wild type or mutant NanoLuc-NRP1. BRET ratios were calculated as fluorescence over luminescence emissions. NanoBRET kinetic experiments were performed at 37°C throughout and required furimazine pre-treatment 5 minutes prior to addition of $VEGF_{165}$a-TMR, $VEGF_{165}$b-TMR or $VEGF_{121}$a-TMR (1nM to 20nM). BRET ratios were then calculated every 30 seconds for up to 120 minutes.

Live Cell Confocal Imaging

HEKT293 cells stably expressing HaloTag-VEGFR2, HaloTag-NRP1 or HaloTag-NRP1 Y297A were seeded 48 hours prior to imaging at 20,000 cells/well in 8-well plates (Nunc Lab-Tek, Thermo Fisher Scientific) pre-coated with poly-D-lysine (0.01mg/ml in PBS), then replaced with serum free DMEM following 24 hours. Cells were treated with 0.5μM membrane impermeant HaloTag Alexa Fluor 488 substrate (Promega Corporation, USA) in HBSS/0.1% BSA for 30 min (37°C). Cells were then washed twice and replaced with HBSS/0.1% BSA prior to incubation with 10nM $VEGF_{165}$a-TMR, $VEGF_{165}$b-TMR or $VEGF_{121}$a-TMR in the dark at 37°C. Cells were imaged live using an LSM710 confocal microscope fitted with a 63x Pan Apochromat oil objective (1.4NA) using Argon488 and Argon 546 laser excitation (3% power), a long pass 540 filter and a pin hole diameter of 1 Airy unit. All images were taken at 1024x1024 pixels per frame with 8 averages.

Fluorescence Correlation Spectroscopy (FCS)

Solution based FCS measurements were performed in Nunc LabTek 8-well chambered coverglasses (Thermo-Fisher Scientific, UK) using a LSM510 NLO Confocor3 microscope equipped with a c-Apochromat 40/1.2NA water immersion objective (Zeiss, Germany). The confocal volume was placed 200μm in solution above the surface of the coverglass. Calibration of beam paths was performed using 20nM Rhodamine 6G (Diffusion coefficient (D) = 2.8 10^{-10} m^2/s; Sigma Aldrich, UK) in high performance liquid chromatography grade water (Chromasolv; Sigma Aldrich) with 488nm and 561nm laser lines using 10x10sec reads. A range of $VEGF_{165}$b-TMR or $VEGF_{121}$a-TMR (2-10nM) solutions were prepared in HBSS/0.1% BSA in the presence or absence of 10mM DTT. DTT containing ligand solutions were preincubated for 30min. FCS recordings were collected with 2 sets of 10x10secs reads using 561nm laser excitation (20% power; AOTF set to 10; equivalent to 0.39kW/cm^2) with fluorescence emissions collected using a long pass 580 (LP580) filter.

Immunofluorescence Labelling

For confocal imaging (Figure S3), HUVECs, wild type HEKT293T cells or HEK293T cells expressing NanoLuc-VEGFR2 or NanoLuc-NRP1 were seeded onto poly-D-lysine coated high resolution coverslips (Zeiss, Germany; 18mmx18mm, 1.5H) at 300,000 cells/well and grown in 6 well culture plates 24 hours prior to experimentation. On the day of the assay, coverslips were transferred to humidified wells lined with parafilm and PBS to avoid dryness and washed 3x5min with PBS. Cells were fixed with 3% paraformaldehyde (PFA)/PBS for 20min at room temperature (RT), washed (3x5min PBS) and incubated with 3% BSA/1% glycine/PBS to reduce non-specific binding (30mins, RT). After washing, cells were blocked with 4% chick serum or donkey serum for VEGFR2 and NRP1 staining respectively (PBS, 30min, RT). This was then replaced with primary antibody diluted 1:200 in 4% serum/PBS and incubated overnight at 4°C (anti-VEGFR1 mAb produced in mice, Sigma V4762; anti-VEGFR2 mAb (mouse), Sigma V9134; anti-NRP1 goat pAb Santa Cruz 7239). The following day, cells were washed and incubated in the dark with secondary antibody diluted 1:500 in 4% serum/PBS for 1 hour at room temperature (VEGFR1 and VEGFR2 chick anti-mouse AlexaFluor488, Invitrogen A21463; NRP1 donkey anti-goat AlexaFluor546, Invitrogen A11056). Coverslips were washed, mounted onto slides using ProLong Diamond (Thermo Fisher Scientific), sealed and stored at 4°C. Coverslips were imaged using a Confocal Zeiss LSM880 fitted with a 63x Pan Apochromat oil objective (1.4NA) using Argon488 or DPSS561 laser excitation at 2% laser power with a pinhole diameter of 1 Airy unit.

To quantify relative receptor expression (Figure S4), HUVECs, wild type HEK293T cells or HEK293T cells expressing NanoLuc- and HaloTag- labelled VEGFR2 or NRP1 were seeded at 25,000 cells/well in black 96-well plates pre-coated with poly-D-lysine

(0.01mg/ml in PBS) and grown for 24 hours (37°C/5% CO_2). Cells were fixed with 3% PFA/PBS then followed an identical immunofluorescence staining protocol as above in 96-well plates with VEGFR2 mouse mAb (Sigma V9134) and NRP1 goat pAb (Santa Cruz 7239). Having labelled with respective secondary antibodies, cells were washed with PBS, nuclei were stained with 2mg/ml H33342 (15 minutes, RT), washed and stored in PBS at 4°C. Cells were imaged using an ImageXpress Micro widefield platereader with a 20x objective at 4 sites per well, with a FITC or TRITC filter for VEGFR2 or NRP1 respectively (500ms exposure time) and a DAPI filter imaging nuclei (25ms exposure time).

QUANTIFICATION AND STATISTICAL ANALYSIS

Data Analysis

All data are presented as mean ± S.E.M. and were analysed using GraphPad Prism 7.02 (San Diego, CA, USA). Equilibrium binding and functional assays were analysed as described in Kilpatrick et al. (2017). A power calculation was performed to confirm sample number for statistical comparisons of pK_i values obtained with different fluorescent ligands. This was done on the basis of 5 separate experiments with the anticipated standard deviation obtained in similar experiments and a calculation of the statistical power to detect a significant change of pK_i of 0.3 log units. This yielded a power of 0.99, i.e. there was a 99% chance of detecting a significant change in pK_i value of 0.3 log units. Statistical analyses using one-way ANOVA are described in the corresponding figure legends or within the text. Significance was defined as $p<0.05$.

High Content Imaging

Images obtained with the ImageXpress Micro widefield platereader at 4 sites per well were quantified using MetaXpress 2.0 (Molecular Devices, USA). Nuclei were quantified with diameter 5-25μm and 100 graylevel intensity above background. VEGFR2 phosphorylation was quantified (Figures 1D and 1E) using a granularity algorithm, granules were defined as 6-12μm diameter with a graylevel intensity of 50 above background. Granularity was quantified per cell, baseline-corrected to non-specific binding (secondary antibody only) and normalised to cediranib-treated wells (0%) and response to 30nM $VEGF_{165}a$ (100%). Quantifying relative receptor expression (Figure S4) using a multiwavelength cell scoring algorithm, regions were defined as 2-15μm in size. Due to distinctions in secondary antibodies, VEGFR2 (FITC) was defined as intensity over 200 graylevels and NRP1 (TRITC) over 50 graylevels. Fluorescence was quantified as integrated intensity per cell and baseline-corrected per experiment to non-specific fluorescence (secondary antibody only).

FCS Autocorrelation Analysis

Autocorrelation analysis was performed using Zen 2010 software (Zeiss, Germany) with all traces fit using a single one component, free 3D Brownian diffusion model, with a pre-exponential included to account for the triplet state of the fluorophore.

DATA AND SOFTWARE AVAILABILITY

GraphPad Prism 7.02 (San Diego, CA, USA) was used to analyse the quantified data and produce the graphs. Zen 2010 (Zeiss; Germany) was used to perform autocorrelation analysis for FCS. MetaXpress 2.0 (Molecular Devices, USA) was used to quantify VEGFR2 phosphorylation and receptor expression labelled with immunofluorescence following high content imaging on the widefield platereader.

Cell Chemical Biology, Volume *25*

Supplemental Information

Real-Time Ligand Binding of Fluorescent VEGF-A Isoforms that Discriminate between VEGFR2 and NRP1 in Living Cells

Chloe J. Peach, Laura E. Kilpatrick, Rachel Friedman-Ohana, Kris Zimmerman, Matthew B. Robers, Keith V. Wood, Jeanette Woolard, and Stephen J. Hill

Supplementary Information.

Real-time ligand binding to VEGFR2 and Neuropilin-1 with fluorescent VEGF-A isoforms that discriminate between the two cell surface proteins in living cells

Chloe J. Peach[1,2,♯], Laura E. Kilpatrick[1,2,♯], Rachel Friedman-Ohana[3], Kris Zimmerman[3], Matthew B. Robers[3], Keith V. Wood[3], Jeanette Woolard[1,2,*], Stephen J. Hill[1,2,*]

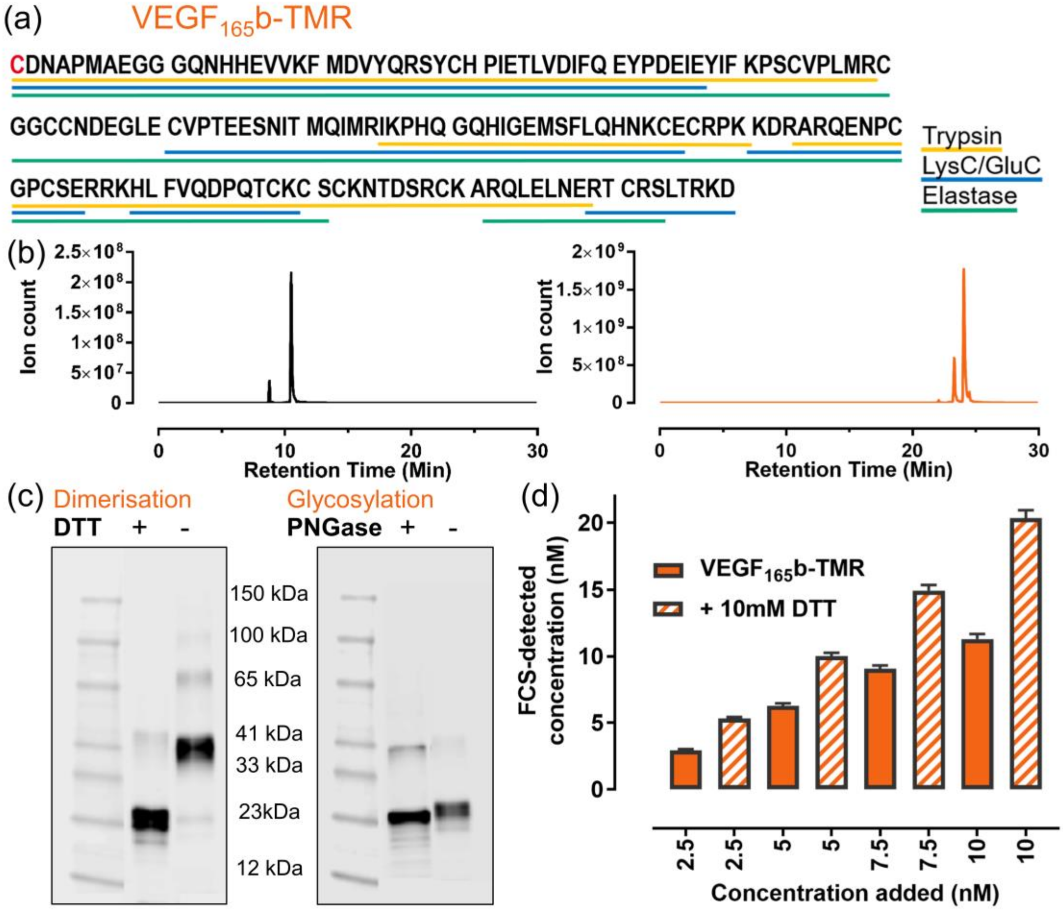

Figure S1. LC-MS/MS analysis of VEGF$_{165}$b-TMR digested with multiple proteases. (Relates to Figure 1). (a) Peptide coverage achieved by digestion with trypsin, LysC/GluC and elastase proteases. The N-terminal cysteine is marked in red. None of the other 14 residues presented in the VEGF$_{165}$b protomer were labelled. Protein identity was confirmed by searching the MS/MS spectra using the Mascot search engine (Matrix Science Inc, Boston, USA) against a human database (SwissProt). The highest scoring hit was the VEGF sequence. (b) LC-MS/MS analysis of the peptide containing the N terminal cysteine (CDNAPMAEGGGQNHHEVVK) derived from VEGF$_{165}$b-TMR (right panel) and VEGF$_{165}$b (left panel) that were purified in the same manner and digested with trypsin protease. Retention times and mono-isotopic masses are given in Supplementary Table 1. (c) Fluorescence SDS-PAGE analysis of VEGF$_{165}$b-TMR (E_{ex}=532 nm; E_{em}=580 nm) in the presence or absence of 100mM DTT and with or without deglycosylation by PNGase. (d) VEGF$_{165}$b-TMR concentration determined using fluorescence correlation spectroscopy (FCS) in the presence or absence of 10mM DTT. N=4-6 separate experiments.

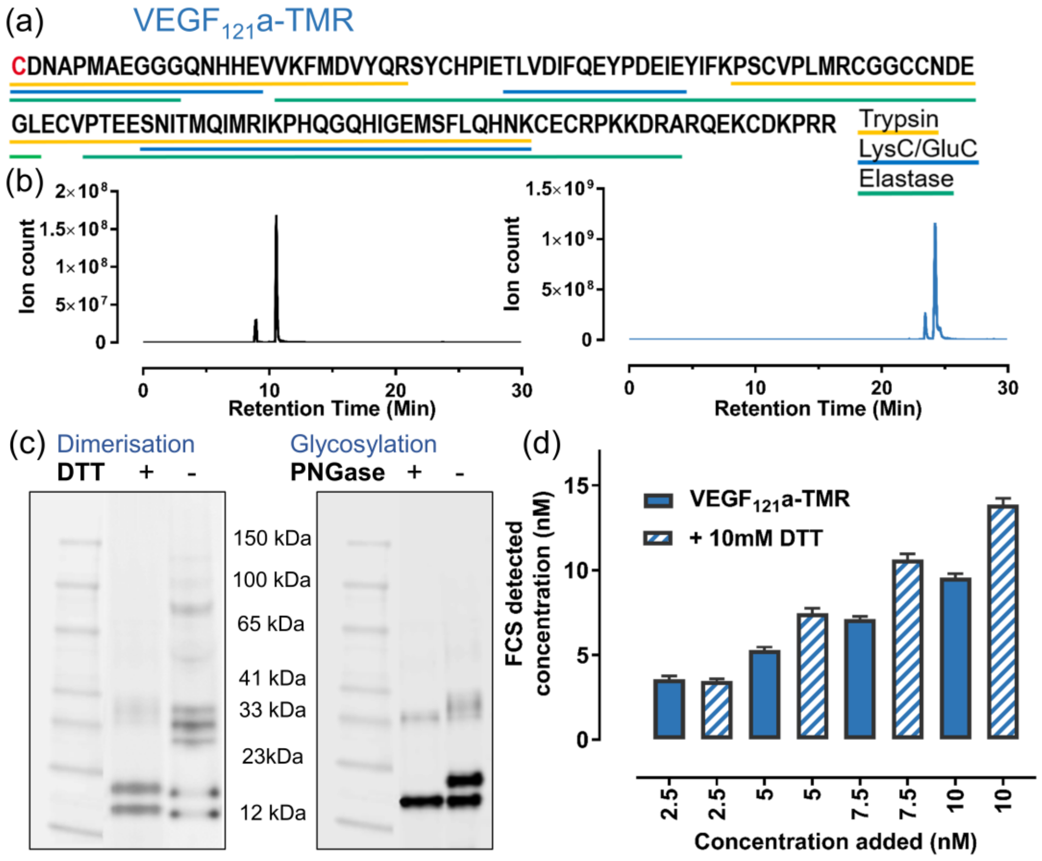

Figure S2. LC-MS/MS analysis of VEGF$_{121}$a-TMR digested with multiple proteases. (Relates to Figure 1). (a) Peptide coverage achieved by digestion with trypsin, LysC/GluC and elastase proteases. The N-terminal cysteine is marked in red. None of the other 8 residues presented in the VEGF$_{121}$a protomer were labelled. Protein identity was confirmed by searching the MS/MS spectra using the Mascot search engine (Matrix Science Inc, Boston, USA) against a human database (SwissProt). The highest scoring hit was the VEGF sequence. (b) LC-MS/MS analysis of the peptide containing the N terminal cysteine (CDNAPMAEGGGQNHHEVVK) derived from VEGF$_{121}$a-TMR (right panel) and VEGF$_{121}$a (left panel) that were purified in the same manner and digested with trypsin protease. Retention times and mono-isotopic masses are given in Supplementary Table 1. (c) Fluorescence SDS-PAGE analysis of VEGF$_{121}$a-TMR (E_{ex}=532 nm; E_{em}=580 nm) in the presence or absence of 100mM DTT and with or without deglycosylation by PNGase. (d) VEGF$_{121}$a-TMR concentration in the presence or absence of 10mM DTT quantified using fluorescence correlation spectroscopy (FCS). N=3-4 separate experiments.

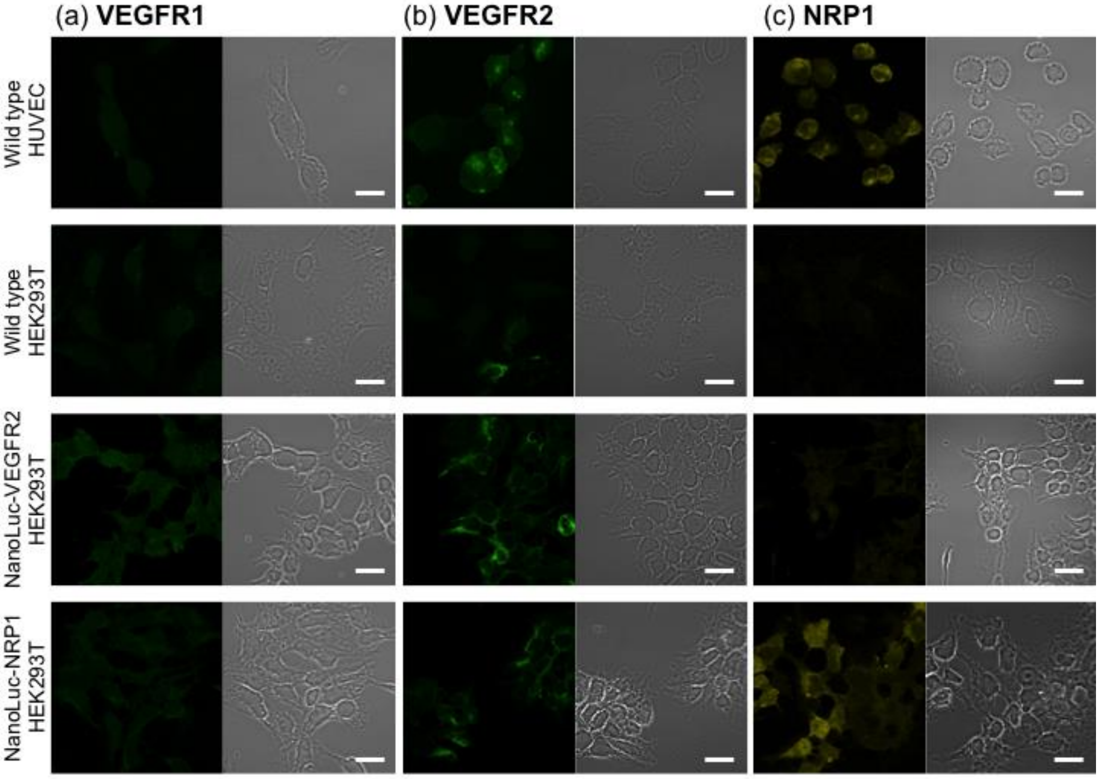

Figure S3. Immunofluorescence labelling of VEGFR1, VEGFR2 and Neuropilin-1 (NRP1) expression in HEK293 cells and HUVECs. (Relates to Figure 2). Immunofluorescence staining for either (A) VEGFR1 (green), (B) VEGFR2 (green) or (B) Neuropilin-1 (yellow), alongside phase contrast images, in fixed cells on coverslips imaged using the Zeiss Confocal LSM880 (63X magnification). Scale bars shown as 20μm and images are representative of n=3.

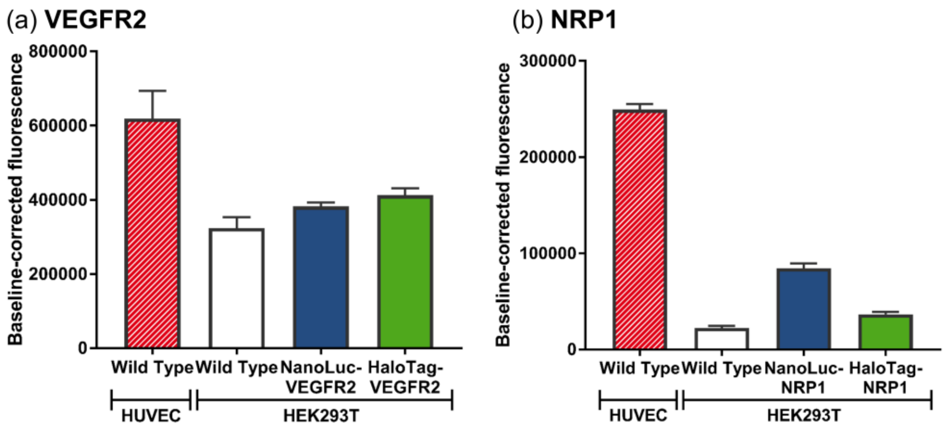

Figure S4. Relative expression of VEGFR2 and NRP1 in endothelial and transfected cell lines. (Relates to Figure 2). Quantified immunofluorescence staining for (A) VEGFR2 or (B) Neuropilin-1, in fixed HUVEC or HEK293T cells imaged in 96-well plates using ImageXpress Micro (20x magnification). Fluorescence was quantified on a per cell basis and baseline-corrected for non-specific fluorescence per experiment (secondary antibody only). Data shown as mean±S.E.M. from 5 independent experiments.

Table S1. (Relates to Figure 1).

LS-MS/MS profiles for unlabelled peptide fragments obtained following protease digestion of VEGF or VEGF-TMR.

**Peptide sequence**	**Digest**	**VEGF isoform**	**Mass/charge (m/z)**	**Charge (z)**	**Mass [M+H]***	**Retention Time (Min)**	**Peak Area**	**Sample**	**%**	**% Labelled**
CDNAPMAEGGGQNHHEVVK	Trypsin	$VEGF_{165}b$	498.97	4	1992.89	10.5	1.36E+08	Labelled	2.9	97.1
CDNAPMAEGGGQNHHEVVK	Trypsin	$VEGF_{165}b$	498.97	4	1992.89	10.5	4.66E+09	Unlabelled	100	n/a
CDNAPMAEGGGQNHHEVVK	Trypsin	$VEGF_{121}a$	498.97	4	1992.89	10.5	6.20E+08	Labelled	6.1	93.9
CDNAPMAEGGGQNHHEVVK	Trypsin	$VEGF_{121}a$	498.97	4	1992.89	10.3	1.01E+10	Unlabelled	100	n/a
CDNAPMAE	LysC/GluC	$VEGF_{165}b$	425.66	2	850.31	13.7	5.26E+06	Labelled	3.5	96.5
CDNAPMAE	LysC/GluC	$VEGF_{165}b$	425.66	2	850.31	13.6	1.52E+08	Unlabelled	100	n/a
CDNAPMAE	LysC/GluC	$VEGF_{121}a$	425.66	2	850.31	13.5	1.14E+07	Labelled	1.2	98.8
CDNAPMAE	LysC/GluC	$VEGF_{121}a$	425.66	2	850.31	13.6	9.23E+08	Unlabelled	100	n/a

LS-MS/MS profiles for unlabelled peptide fragments obtained following protease digestion of VEGF or VEGF-TMR. The labeling efficiency of $VEGF_{165}b$-TMR or $VEGF_{121}a$-TMR was determined by comparing the integrated peak areas of the unmodified proteolytic peptides in the labelled and unlabelled samples that were digested with trypsin or LysC/GluC. The analysis indicated 94-99% labelling efficiency. *Mono-isotopic mass at a single charge assuming the peptide takes up a single proton. Mono-isotopic mass [M+H] is calculated as (m/z)*z – (z-1).

Table S2 (relates to Figure 1).

LS-MS/MS profiles for labelled peptide fragments obtained following protease digestion of VEGF or VEGF-TMR.

**Peptide sequence**	**Digest**	**VEGF isoform**	**Mass/charge (m/z)**	**Charge (z)**	**Mass of labelled peptide [M+H]***	**Mass of unlabelled peptide [M+H]***	**Mass Difference**
CDNAPMAEGGGQNHHEVVK	Trypsin	$VEGF_{165}b$	937.39	3	2810.16	1992.89	817.27
CDNAPMAEGGGQNHHEVVK	Trypsin	$VEGF_{121}a$	937.39	3	2810.16	1992.80	817.27
CDNAPMAE	LysC/GluC	$VEGF_{165}b$	834.30	2	1667.59	850.31	817.28
CDNAPMAE	LysC/GluC	$VEGF_{121}a$	834.30	2	1667.59	850.31	817.28

LS-MS/MS profiles for labelled peptide fragments obtained following protease digestion of VEGF or VEGF-TMR. Observed mass of the labelled peptides containing the N-terminal cysteine confirmed a mass increase of 817 Da due to covalent attachment of the 6-TMR-PEG-CBT. *Mono-isotopic mass at a single charge assuming the peptide takes up a single proton. *Mono-isotopic mass [M+H] is calculated as (m/z)*z – (z-1).

Table S3. Competition binding pK_i values for VEGF-A isoforms binding to VEGFR2 and NRP1 (relates to Figures 3 and 4).

	**NanoLuc-VEGFR2**	**NanoLuc-VEGFR2**	**NanoLuc-VEGFR2**	**NanoLuc-NRP1**
**VEGF isoform**	**$VEGF_{165}$a-TMR**	**$VEGF_{165}$b-TMR**	**$VEGF_{121}$a-TMR**	**$VEGF_{165}$a-TMR**
VEGF-Ax	9.20 ± 0.02	9.57 ± 0.03	9.65 ± 0.06	ND
$VEGF_{165}$a	9.57 ± 0.04	9.73 ± 0.09	9.54 ± 0.05	9.54 ± 0.21
$VEGF_{165}$b	9.07 ± 0.02	9.61 ± 0.06	9.28 ± 0.10	ND
$VEGF_{121}$a	9.30 ± 0.06	9.58 ± 0.18	9.31 ± 0.08	ND
$VEGF_{145}$a	8.83 ± 0.03	8.92 ± 0.08	8.82 ± 0.08	7.82 ± 0.15
$VEGF_{189}$a	9.13 ± 0.03	8.92 ± 0.03	9.06 ± 0.09	8.60 ± 0.14
$VEGF_{111}$a	9.66 ± 0.13	9.89 ± 0.15	9.98 ± 0.05	ND

Summary of binding affinities (pK_i) of unlabelled VEGF-A isoforms determined from inhibition of the binding of $VEGF_{165}$a-TMR, $VEGF_{165}$b-TMR or $VEGF_{121}$a-TMR to NanoLuc-VEGFR2 and NanoLuc-NRP1. Data are expressed as mean ± S.E.M determined from 5 independent experiments. ND = not determined due to lack of significant inhibition of binding with 30nM unlabelled VEGF isoform.
